# Mean-field and Fluctuations for Hub Dynamics in Heterogeneous Random Networks

**DOI:** 10.1007/s00220-025-05335-0

**Published:** 2025-06-23

**Authors:** Zheng Bian, Jeroen S. W. Lamb, Tiago Pereira

**Affiliations:** 1 Instituto de Ciências Matemáticas e de Computação, Universidade de São Paulo, 13566-590 São Carlos, Brazil; 2 Department of Mathematics, Imperial College London, SW7 2AZ London, UK; 3 International Research Center for Neurointelligence, 113-0033 Tokyo, Japan; 4 Centre for Applied Mathematics and Bioinformatics, Department of Mathematics and Natural Sciences, Gulf University for Science and Technology, 32093 Halwally, Kuwait

## Abstract

We study a class of heterogeneous random networks, where the network degree distribution follows a power-law, and each node dynamics is a random dynamical system, interacting with neighboring nodes via a random coupling function. We characterize the hub behavior by the mean-field, subject to statistically controlled fluctuations. In particular, we prove that the fluctuations are small over exponentially long time scales and obtain Berry-Esseen estimates for the fluctuation statistics at any fixed time. Our results provide an explanation for several numerical observations, namely, a scaling relation between system size and frequency of large fluctuations, the system size induced desynchronization, and the Gaussian behavior of the fluctuations.

## Introduction

Network systems are fruitful models for various naturally occurring and man-made systems ranging from neuroscience [BGP09] and physics [MS09] via electrochemistry [NOEE22], to social sciences [SPMS17] to mention a few applications. In the case of homogeneous networks, where symmetries facilitate the analysis, many ergodic and statistical properties of the network system are known, in the context of coupled map lattices [CF05], all-to-all coupled systems [Fer19, BLS23], and in the all-to-all thermodynamic limit [ST21, ST22, Gal22]. Much less is known about heterogeneous networks, another class of realistic models which generally lack symmetry and feature massively connected nodes, referred to as *hubs*, coexisting with poorly connected nodes [JSS13].

Hubs arise persistently in large random heterogeneous networks [BB21] and play an important role in network systems. In addition to regulating the information flow and providing resilience during attacks [AB02], hubs affect the collective dynamics of the network [BGP09]. In fact, hubs may lead to a hierarchical transition toward global synchronization when the isolated dynamics of each node is periodic [GGMA07]. Hubs can induce the optimal collective response of the network to noise [TFP21], an abrupt transition to collective motion [VZP15]. When the isolated dynamics is chaotic, hubs inhibit global synchronization [PvST20] but can spark the onset of cluster synchronization [CBPM23, MC24].

Even when individual interactions are weak, the hub behavior can change due to the collective interaction with its neighbors. Understanding hub behavior is intricate because the network system is high dimensional. Nonetheless, numerical and experimental results suggest that over very long time scales hub dynamics can be well approximated by a low-dimensional system given by the mean-field [Per10, BRS12, Ric16]. When each node dynamics is an expanding map, recent work [PvST20] has proved this dimensional reduction under some restrictive assumptions. Our results address three main shortcomings of previous works:

i) Resilience against local perturbation. Previous work required hyperbolicity for the mean-fields of *all* nodes. This assumption seems unnecessary: since hubs interact with a large number of nodes, the failure of a few nodes should not change the overall hub dynamics.
ii) Networks with power-law degree distribution. Previous results also required the network to feature a degree separation between hubs and low degree nodes. This dichotomy is not present in most networks, where massively connected nodes coexist with other hubs that are not so well connected, leaving no gap between hubs and low degree nodes.
iii) Characterization of large fluctuations. Over given time-scales hubs admit the mean-field approximation up to predominantly small fluctuations. It remains an open problem to statistically characterize the rare occurrences of large fluctuations in terms of network characteristics, such as size and degree distribution.

In this paper, we meet these challenges by exploiting the typicality of random trajectories, and thereby overcoming some major technical challenges arising in the gap between topological dynamics and ergodic theory. We also apply concentration inequalities to the Chung-Lu random power-law network to establish certain graph theoretic properties. In particular, we advance the state-of-the-art of ergodic theory for network dynamics and complex systems by characterizing hub dynamics in random power-law networks, in terms of the mean-field subject to Gaussian-like fluctuations. We exhibit examples of uniformly contracting node dynamics in Introduction and Section 3 and comment on expanding and nonuniformly contracting cases in Remarks 2.5, 3.4.

In the following, we set up the network system in subsection 1.1 and showcase numerical observations on the star network in subsections 1.2.1–1.2.3 and on a power-law network in subsections 1.3.1–1.3.2. In subsections 1.2.4 and 1.3.3, we discuss our main theorems, which formalize the numerical observations. Section 1.2 is dedicated to the case of star network, which is sufficient to explain the essence of the general result without too many technical details. Section 1.3 generalizes to a random power-law graph. In later sections 2.1 and 2.2, we provide abstract versions of the results covering both cases.

### Network random dynamical system

The *network random dynamical system* is the datum $(G,f,h,\alpha )$, where *G* is an undirected graph on $N\ge 2$ nodes, $f=\{f_{\omega }:\mathbb {T}\rightarrow \mathbb {T}\}$ is a family of random circle maps that compose the node dynamics, $h=\{h_{\omega }:\mathbb {T}\times \mathbb {T}\rightarrow \mathbb {R}\}$ is a collection of random coupling functions that describe the pairwise interaction between neighbor nodes in *G*, and $\alpha >0$ is the coupling strength. The graph *G* is represented by the adjacency matrix $A=(A_{ij})_{i,j=1}^N$, where $A_{ij}=1$ if nodes *i*, *j* are connected and 0 otherwise. The degree of node *i* is $k_i:= \sum _{j=1}^N A_{ij}$, and the largest degree $\Delta _0:= \max _{i=1,\cdots ,N} k_i$. The state $x_i^{t+1}$ of node *i* at time $t+1$ is given by

1 $$\begin{aligned} x_i^{t+1} = f_{\varvec{\omega }_i^t}(x_i^t) + \frac{\alpha }{\Delta _0} \sum _{j=1}^{N} A_{ij} h_{\varvec{\omega }_i^t} (x_i^t,x_j^t)\mod 1,~~~~i=1,\cdots ,N. \end{aligned}$$

In the above equation, $\alpha$ is rescaled by $\Delta _0$ so that the most massively connected node receives an order-one interaction.

#### Remark 1.1

*(Notation of noise realization)*. In the introduction, we use the simple font $\omega$ to index the family of circle maps $f_{\omega }$ and coupling functions $h_{\omega }$, and the boldface $\varvec{\omega }=(\varvec{\omega }_i^t)$ to represent a vector of noise realizations with time $t=0,1,\cdots$ and node $i=1,\cdots ,N$ coordinates.

#### Example 1.2

As an example of node dynamics *f*, consider a family of contractions

$f_\omega :\mathbb {T}\rightarrow \mathbb {T}$ on the circle

2 $$\begin{aligned} f_\omega (x):={\left\{ \begin{array}{ll} \frac{x}{2} +\frac{\omega }{4},& x\in [0,\frac{1}{2}]\\ \frac{1-x}{2} +\frac{\omega }{4},& x\in [\frac{1}{2},1] \end{array}\right. }, ~~~~\omega =0,1,2,3, \end{aligned}$$

and iterate the dynamics at each time step by choosing from $\{f_{\omega }:\omega =0,1,2,3\}$ randomly independently and identically with probability 1/4 for each contraction.

**Fig. 1:**
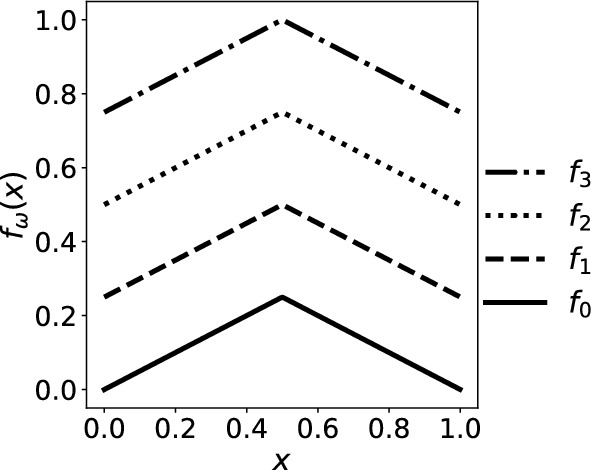
Graphs of four contractions $f_{\omega }$, $\omega =0,1,2,3$ on the circle $\mathbb {T}=[0,1]/0\sim 1$

In this example the node dynamics admit $\textrm{Leb}_{\mathbb {T}}$ as the unique stationary measure. For other choices of $f_{\omega }$, the stationary measure can be absolutely continuous or singular with respect to $\textrm{Leb}_{\mathbb {T}}$, including measures supported on a Cantor set; Theorem 3.3 will cover a more general case.

As an example of random coupling function, consider

3 $$\begin{aligned} h_\omega (x,y)= \sin 2\pi y- \sin 2\pi x - \frac{\omega }{3.6},~~~~\omega =0,1,2,3. \end{aligned}$$

More generally, *h* can be any family of $C^4$ maps $\mathbb {T}\times \mathbb {T}\rightarrow \mathbb {R}$.

In the rest of the Introduction, we continue with the concrete examples of node dynamics $f_{\omega }$ and coupling function $h_{\omega }$ as in Example 1.2.

### Dynamics on the star network

Consider a star graph *G* on $N=10^6$ nodes: one hub together with $L=N-1$ low-degree nodes, where the hub influences each low-degree node, and each low-degree node influences the hub but not the other low-degree nodes. We index the hub by 1 and write *z* for $x_1$, so the star network dynamics reads

4 $$\begin{aligned} \begin{aligned} z^{t+1}&= f_{\varvec{\omega }_1^t}(z^t) + \frac{\alpha }{L} \sum _{j=2}^N h_{\varvec{\omega }_1^t} (z^t,x_j^t)\mod 1,\\ x_j^{t+1}&= f_{\varvec{\omega }_j^t}(x_j^t) + \frac{\alpha }{L} h_{\varvec{\omega }_j^t}(x_j^t, z^t)\mod 1,~~~~j=2,\cdots ,N. \end{aligned} \end{aligned}$$

Here the noise $\varvec{\omega }_i^t$ is assumed to be iid in time $t=0,1,\cdots$ and in node coordinates $i=1,\cdots ,N$, assigning weight 1/4 to each $\omega \in \{0,1,2,3\}$.

#### Mean-field dimensional reduction

**Fig. 2:**
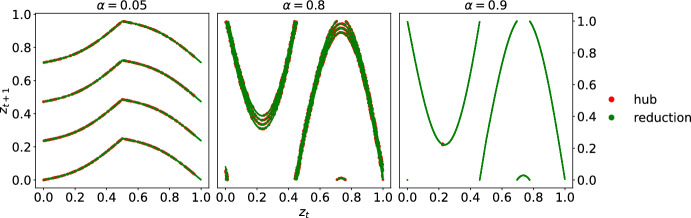
Numerical simulations for the star network dynamics (4) on $N=10^6$ nodes at various coupling strengths $\alpha =0.05, 0.8,0.9$ on the left, center and right panels respectively, with iid random iteration of four circle contractions (2) as isolated node dynamics and (3) as pairwise interaction. The plots show the return behaviors of the hub, that is, the states $z^t$ on horizontal axis against the next states $z^{t+1}$ on vertical axis. Novel hub behaviors emerge from network interactions and vary across different coupling strengths: uniform contraction, expanding region and deterministic fixed point. The mean-field dimensional reduction ansatz yields a reduced one-dimensional system, whose graph, plotted in green, fits very well the actual hub behavior in red

We simulate the star network by probing three coupling strengths $\alpha =0.05$, 0.8, 0.9. For each $\alpha$, we initialize the node states $(z^0,x_2^0,\cdots ,x_N^0)\in \mathbb {T}^N$ at random with uniform distribution in [0, 1). Then, we iterate Eq. (4), discard the first 5000 iterates, and collect the next 1000 iterates. In Figure 2, we plot the hub return map $z^t$ versus $z^{t+1}$ in red. As the coupling strength $\alpha$ varies, the hub behavior differs from the isolated node dynamics. In Figure 1 on the left panel at $\alpha =0.05$, the hub remains contractive, on the central panel at $\alpha =0.8$, the hub dynamics appear to have an expanding region, and lastly, on the right panel at $\alpha =0.9$, the hub appears to hover around a fixed point near 0.2. This shows the variety of hub behaviors emergent from the interactions, according to different coupling strengths. Due to our choice of $h_{\omega }$ in Eq. (3), the randomness in $f_{\alpha ,\omega }$ collapses at $\alpha =0.9$, resulting in the single graph of $f_{0.9}=f_{0.9,\omega }$, $\omega =0,1,2,3$ as shown in the right panel of Figure 2.

From Figure 2, we observe that the emergent hub behavior resembles another one-dimensional random system. Since the low degree nodes $j=2,\cdots ,N$ receive only one contribution from the hub of order $O(L^{-1})$, we expect that the statistics of the low degree node to resemble the unique stationary measure $\textrm{Leb}_{\mathbb {T}}$. In particular, the aggregate effect of the low degree nodes on the hub should be approximated by a space average

$$\begin{aligned} \frac{1}{L}\sum _{j=2}^N h_{\varvec{\omega }_1^t}(z^t,x_j^t)&\approx \int _{\mathbb {T}} h_{\varvec{\omega }_1^t}(z^t,x)\textrm{d}x \\&= \sin 2\pi z^t - \frac{\varvec{\omega }_1^t}{3.6} \end{aligned}$$

taken against the Lebesgue measure on the circle, which is the unique stationary measure of the isolated node dynamics and captures its typical statistics; see Theorem 3.3. This ansatz, referred to as the *mean-field dimensional reduction*, approximates the hub behavior by a one-dimension system

$$ z^{t+1} = f_{\alpha , \varvec{\omega }_1^t}(z^t) + \xi ^t(\varvec{\omega },x), $$

where the *mean-field reduced map* reads

5 $$\begin{aligned} f_{\alpha , \omega }(z):= f_{\omega }(z) + \alpha \int _{\mathbb {T}} h_{\omega }(z, x)\textrm{d}x\mod 1, \end{aligned}$$

and the *mean-field fluctuation* at time *t* from initial datum $(\varvec{\omega } ,x)$ is

$$ \xi ^t(\varvec{\omega },x): = \frac{\alpha }{L} \sum _{j=2}^N \sin 2\pi x_j^t. $$

Such approximation is meaningful when the fluctuation $| \xi ^t | \ll 1$. In Figure 2, we plot in green the graph of this one-dimensional system, and the actual hub behavior in red, numerically corroborating the mean-field dimensional reduction. Theorem A (i) provides the corresponding mathematical statement, proving the reduction.

#### Frequency of large fluctuations

To illustrate the impact of system size *L* on fluctuations $\xi ^t$, we simulate for each *L* the star network dynamics at coupling strength $\alpha =0.9$ for *T* iterations, and count the number

$$ n^T_{\varepsilon }= \#\left\{ t<T: |\xi ^t|>\varepsilon \right\} $$

of times up to *T* that the fluctuation exceeds a fixed threshold $\varepsilon$. Then we calculate the frequency $\rho _{\varepsilon }^T$ of large $(>\varepsilon )$ fluctuations up to time *T*

$$\rho _{\varepsilon }^T = n^T_{\varepsilon }/T.$$

**Fig. 3:**
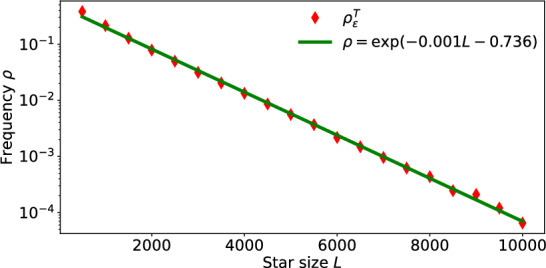
Frequency of large fluctuations decreases exponentially in system size. The red diamonds mark the frequency $\rho _{\varepsilon }^T$ up to time $T=2\times 10^5$ of large mean-field fluctuations, i.e., $|\xi ^t|>\varepsilon$ with threshold $\varepsilon =0.025$. The horizontal axis for system size *L* is in linear scale, whereas the vertical axis for frequency $\rho _{\varepsilon }^T$ is in logarithmic scale. The green line provides a tight linear fit, indicating an exponential decrease of $\rho _{\varepsilon }^T$ in *L*

In our simulations, we fix threshold $\varepsilon = 0.025$, vary the star size *L* from 500 to $10^4$ in steps of 500, and simulate each system for a total time $T=2\times 10^5$. In Figure 3, we show this frequency $\rho _{\varepsilon }^T$ versus *L* in red diamonds. The green line is a linear fit of $\log \rho _{\varepsilon }^T$ against *L*, which strongly suggests that

$$ \rho _{\varepsilon }^T ~= A e^{-\gamma L} ,~~~~A=e^{-0.736}, ~~\gamma =0.001 $$

and the chance to see the departure of the dynamics from mean-field reduced map becomes exponentially small in *L* as the size *L* of the star network grows. For quantitative relations between system size *L* and frequency of large fluctuations $|\xi ^t|>\varepsilon$, see Theorem A (ii) below.

#### Gaussian behavior of the fluctuations

**Fig. 4:**
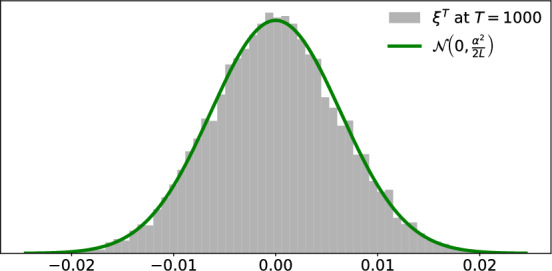
Gaussian fluctuations. The grey histogram presents the fluctuations data $\{\xi _n^T\}_{n=1}^{10^4}$ corresponding to $10^4$ independent trials of network initial conditions; each $\xi _n^T$ is obtained by starting at initial condition trial *n* and iterating for $T=1000$ times the network dynamics at coupling strength $\alpha =0.9$ on the star of size $L=10^4$. The green curve shows the probability density function of the normal distribution $\mathcal {N}(0,\frac{\alpha ^2}{2L})$ with zero mean and variance $\frac{\alpha ^2}{2L}$. The tight fit indicates that the fluctuation $\xi ^T$ at time $T=1000$ has Gaussian statistics

The fluctuation $\xi ^t$ can be interpreted as an ensemble average of the low degree node states through the observable $x\mapsto \alpha \sin 2\pi x$. The low degree nodes are almost isolated, up to hub influence of order $O(L^{-1})$, and hence almost independent from each other. Hence, we expect the fluctuations $\xi ^t$ to follow a Central Limit behavior. To illustrate this, we fix star size $L=10^4$ and coupling strength $\alpha =0.9$, and take $10^4$ trials of network initial conditions randomly independently and uniformly in $\mathbb {T}^{L+1}$. For each trial *n* we simulate the star network dynamics up to time $T=1000$ and calculate the terminal fluctuation $\xi ^T_n$. We plot the histogram of the data $\{ \xi ^T_n \}_{n=1}^{10^4}$ in Figure 4. Superimposed in green is the probability density function of the normal distribution $\mathcal {N}(0,\frac{\alpha ^2}{2L})$ with zero mean and variance $\frac{\alpha ^2}{2L}$. The close fit indicates that $\mathcal {N}(0,\frac{\alpha ^2}{2L})$ indeed captures the fluctuation statistics at time *T*; see Theorem A (iii).

#### Statement of main result on the star network

Theorem A below underpins the observations made for the star network dynamics (4): the mean-field dimensional reduction for hub behavior in Section 1.2.1 is addressed in item (i), scaling relations for the large fluctuation frequency in Section 1.2.2 are addressed in item (ii), and finally, the Gaussian nature of fluctuations in Section 1.2.3 are addressed in item (iii).

In the statement below, we use $\textrm{Prob}$ to denote the product probability measure on $\Omega \times \mathbb {T}^N$ of the Bernoulli measure for $\varvec{\omega }\in \Omega =\{0,1,2,3\}^{\mathbb {N}\times N}$ times the volume for $x\in \mathbb {T}^N$.

##### Theorem A

$\mathbf {Hub\,Dynamics\,in\,Star\,Networks}$**.** Consider the dynamics (4) on a star network with $f_{\omega },h_{\omega }$ as in Example 1.2, and initial conditions following the uniform distribution on $\mathbb {T}^N$, $N=L+1\gg 1$. Then, at coupling strength $\alpha \ll L^{1/2}$, the hub evolution admits mean-field dimensional reduction defined in (5), namely:

$$\mathrm {(i)}$$
**Almost sure reduction in asymptotic time:** for any $\varepsilon \gg \alpha L^{-1/2}$,
  $$ \textrm{Prob} \left\{ (\varvec{\omega },x): \liminf _{T\rightarrow +\infty }\frac{1}{T}\sum _{t=0}^{T-1} \mathbbm {1}_{|\xi ^t(\varvec{\omega },x)| \le \varepsilon } \ge 1-\exp (-L \varepsilon ^2 \alpha ^{-2}/9)\right\} =1; $$
$$\mathrm {(ii)}$$
**Small fluctuation in long time windows:** in time windows
  $$I_{t_0}^T:=\{t_0,\cdots ,t_0+T-1\},~~~~T\ge \exp (L^{1-2\kappa }),~~t_0\in \mathbb {N},$$
  we have successively small fluctuations
  $$ \textrm{Prob}\left\{ (\varvec{\omega },x): \max _{t\in I_{t_0}^T} |\xi ^t(\varvec{\omega },x)| \le 3L^{-\kappa } \alpha \right\} \ge 1-\exp (-L^{1-2\kappa }),~~~~\kappa \in (0,1/2); $$
$$\mathrm {(iii)}$$
**Gaussian fluctuations: **at any time $t\in \mathbb {N}$, the fluctuation $\xi ^t$ is approximately Gaussian, i.e.,
  $$ \textrm{Prob}\left\{ (\varvec{\omega },x): \xi ^t(\varvec{\omega },x) \le s\right\} \in \left[ F_L\left( s -c_1\right) -c_2, F_L\left( s+c_1 \right) +c_2\right] ,~~~~s\in \mathbb {R},$$
  where $F_L$ denotes the cdf of the normal distribution with zero mean and variance $\alpha ^2/(2L)$, and the correction constants are
  $$ c_1=36\alpha ^2 L^{-1},~~~~c_2=8 L^{-1/2}. $$

##### Remark 1.3

To state the relations among $L,\alpha ,\varepsilon$ more precisely in Theorem A, we mean that there are constants $C_1,C_2,C_3>0$ such that if $L\ge C_1$, $L^{1/2}/\alpha \ge C_2$ and $\varepsilon L^{1/2}/\alpha \ge C_3$, then Items (i), (ii) and (iii) hold.

In Item (i), $\mathbbm {1}_{|\xi ^t(\varvec{\omega },x)| \le \varepsilon }$ indicates whether or not the fluctuaion $\xi ^t(\varvec{\omega },x)$ at time *t* is small; the time average computes the relative frequency of small fluctuations in the time window $t=0,\cdots ,T-1$; by passing to the limit inferior we obtain the asymptotic frequency of small fluctuations starting from initial data $(\varvec{\omega },x)$; finally, Item (i) says that with full probability, the asymptotic frequency of small fluctuations is close to one.

In Items (i) and (ii), we have provided an upper bound for the fluctuation size and a lower bound for the asymptotic frequency; the constants $A,\gamma$ in Figure 3 are not sharp and generally may depend on the coupling function. Theorem A is a consequence of the more general Theorem 2.4 and the uniform typicality of the random orbits of the contractions (2), see Theorem 3.3. The derivation of the explicit constants is discussed in Appendix A.1. We briefly discuss the proof strategy in this particular case, which will become a fundamental step in Theorem 2.18 for dimensional reduction on more complex networks.

**Main ideas of proof for Theorem A.** For item (i) our strategy follows three steps:

1. We recast the dimensional reduction into a problem about visits to the so-called bad set, i.e., a region in the state space $\mathbb {T}^N$ that produces large fluctuation $\xi ^t$.
2. We show that the bad set has a small size, according to large deviation results. By ergodicity, the frequency of such visits by a typical isolated orbit is as small as the size of the bad set.
3. We relate the low-degree node orbit to the isolated orbit by shadowing. The major challenge is to bridge the fundamental gap between the topologically constructed shadowing orbit and typicality in the ergodic sense. Our Theorem 2.4 treats the general case assuming compatibility of shadowing and ergodicity. In Section 3 this compatibility is verified for the case of iid random iteration of contractions as node dynamics.

Steps 1 and 2 were put forward in [PvST20] and adapted to our setting. Our contribution in step 3 concerns the *ergodic* properties of the shadowing orbit, a *topologically* constructed object; this problem is difficult and generally open, see Remark 2.5. We resolve this problem in Theorem A by using *Breiman’s ergodic theorem* together with the *uniform contraction* property to ensure the typicality of random orbits for almost every noise realization independent of the initial condition. This concludes item (i).

Item (ii) builds on step 3. By choosing the fluctuation threshold $\varepsilon =O(L^{-\kappa })$ for some $\kappa \in (0,1/2)$, we obtain an estimate for the size of bad set, which, by stationarity of the isolated random system, equals the probability that the typical isolated orbit hits the bad set at any time. The estimates follow by excluding the probability of these bad hits for each time in a consecutive window.

Item (iii) follows from Berry-Esseen estimates together with our shadowing technique in step 3. We consider the isolated node dynamics, observed through $x\mapsto \sin 2\pi x$, as iid random variables on $\Omega \times \mathbb {T}^{N}$. The fluctuation $\xi ^t$ is thus the ensemble average, whose Gaussian nature conforms to the classic Berry-Esseen estimates. Our result follows by comparing orbit-wise the isolated dynamics to the low degree trajectory as in step 3.

**Resilience against local perturbation.** Consider a minor malfunction in the star network dynamics of one low degree node, which switches to non-hyperbolic behavior. Our reduction technique still decouples the other low degree nodes into typical shadowing orbits and obtains the same control on the fluctuation, up to an $O(L^{-1})$ loss due to the malfunctioning low degree node.

Another major advantage of this resilience of our technique is the generalizability to dynamics on more realistic networks that feature a power-law degree distribution. An important feature of many real-world networks is the power-law degree distribution, that is, the frequency *P*(*k*) of nodes of degree *k* in the network is proportional to $k^{-\beta }$ for some power-law exponent $\beta >0$. Internet, World Wide Web, and power grids are known to have power-law degree distribution [CL06]. The reduced equation 5 depends on the effective coupling strength $\alpha _i$, which is determined by $\alpha$ as well as the hub degree; see Theorem B below. The node degrees in the intermediate range give rise to a continuum of dynamical possibilities between the massively connected hub behavior and the almost isolated behavior. Nodes of a certain intermediate degree are bound to lose hyperbolicity in their mean-field reduced behavior, violating the global hyperbolicity assumption.

Our technique enables us to obtain dimensional reduction principle for realistic networks without gaps in degree distribution, such as power-law networks. In fact, many other networks are covered by our result, as long as the locally star-like property is satisfied, see Sections 2.2 and 3.2.

### Dynamics on power-law networks

**Fig. 5:**
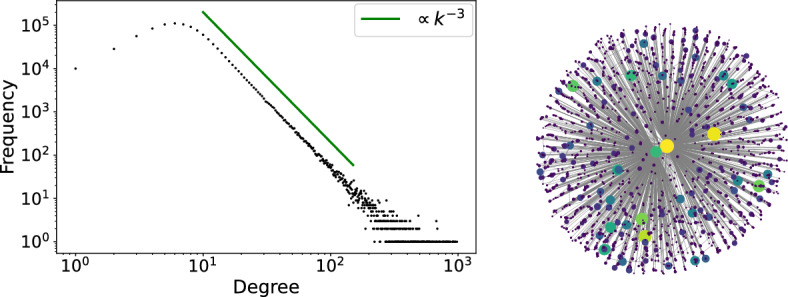
Random power-law network $G_1$ generated from Chung-Lu model on $N=998168$ nodes with power-law exponent $\beta =3$, largest degree $\Delta _0=979$ and lowest degree 1. The left panel shows in log-log scale the degree distribution of $G_1$, that is, degree *k* in horizontal axis versus the frequency *P*(*k*) of nodes of degree *k*. The power-law in green highlights the fact that $P(k)\propto k^{-3}$. The right panel draws the subgraph *S* of $G_1$ restricted to three nodes of degrees 54, 875, 979, shown in the center, together with their neighbors in $G_1$ shown as surrounding, with node degrees reflected by size and color. This indicates that most neighbors of a hub in $G_1$ are of low degree

We use the Chung-Lu model [CL06] to produce large power-law networks with well-understood graph-theoretic properties. To construct a connected random power-law graph $G_1$, we first construct a Chung-Lu random graph $G_0$ from expected degree sequence

6 $$\begin{aligned} \begin{aligned} w_i&:= \frac{\beta -2}{\beta -1} w n^{\frac{1}{\beta -1}} \left[ n\left( \frac{w(\beta -2)}{m(\beta -1)}\right) ^{\beta -1} +i-1\right] ^{-\frac{1}{\beta -1}},~~~~i=1,\cdots ,n, \end{aligned} \end{aligned}$$

where $n=10^6$ denotes the number of nodes, $\beta =3$ power-law exponent , $w=10$ average expected degree, and $m=10^3$ largest expected degree. On the empty graph consisting of nodes $1,\cdots ,n$, we add links between nodes *i* and *j* as independent Bernoulli variables with success probability $p_{ij} = w_iw_j/\sum _{k=1}^nw_k$. The resulting graph $G_0$ is not connected, but has a giant component, which we take to be $G_1$. By concentration inequalities, we will see in Lemma 3.6 that the actual degree sequence $k_i$ is concentrated at the expected version $w_i$ above; moreover, we will prove that this random power-law graph is locally star-like in the sense that most neighbors of any hub are of low degree, see Theorem B. In our simulation, $G_1$ consists of $N=998168$ nodes, with maximum degree $\Delta _0=979$ and minimum degree 1, and is a connected power-law graph. To showcase its power-law degree distribution, we plot in Figure 5 left panel the degree *k* against the frequency *P*(*k*) of nodes of degree *k* in log-log scale. For comparison, we show a power-law $k^{-3}$ in green.

**Hubs** $\mathcal {H}_{\Delta }$, **low degree nodes** $\mathcal {L}_{\delta }$, **and the star-like index** $\nu$. In $G_1$ most neighbors of any hub *i* are low degree nodes; i.e., the power-law graph is locally star-like. To illustrate, we draw a subgraph of $G_1$ by selecting three nodes of degrees 54, 875 and 979 respectively, shown in the center of the right panel in Figure 5, together with their neighbors shown as surrounding. The colors and sizes of the nodes reflect their degrees in $G_1$.

Figure 5 left panel shows no gap in the degree distribution; in particular, there are no natural scales to distinguish the hubs from low degree nodes, so we have to introduce them by hand. For $G_1$, we put hub scale $\Delta =900$, low degree scale $\delta =100$, and thus define the collection of $\Delta$-*hubs* to be

$$\mathcal {H}_{\Delta }:=\left\{ i: k_i>\Delta \right\} $$

and the collection of $\delta$-*low degree nodes* to be

$$\mathcal {L}_{\delta }:= \left\{ j: k_j< \delta \right\} ;$$

we find $M:=\#\mathcal {H}_{\Delta }=7$ hubs and $L:=\#\mathcal {L}_{\delta }=995635$ low degree nodes in $G_1$. More generally, the choice of these thresholds $\Delta ,\delta$ is a delicate issue and will be treated in detail later. Roughly speaking, a hub is understood as any node *i* whose degree $k_i$ is comparable with the largest degree $\Delta _0$, whereas a low degree node *j* has $k_j/\Delta _0 \rightarrow 0$ as $\Delta _0$ grows. Denote by $\mathcal {N}_i:=\{j: A_{ij}=1\}$ the set of neighbors of node *i*. We define the *star-like index* $\nu _i$ *at hub* $i\in \mathcal {H}_{\Delta }$ to be the proportion of low degree neighbors $\mathcal {N}_i\cap \mathcal {L}_{\delta }$ of hub *i*

$$\nu _i:= \frac{\#\mathcal {N}_i\cap \mathcal {L}_{\delta }}{ k_i},$$

and the *star-like index* $\nu$ *of network* $G_1$ to be the minimum star-like index among all hubs

$$ \nu := \min \{\nu _i:i\in \mathcal {H}_{\Delta }\}. $$

In $G_1$, we find $\nu =0.941$; in other words, more than $94.1\%$ of neighbors of each of the seven hubs in $G_1$ are of low degree. As we will prove in Theorem 3.7, the star-like index $\nu$ of a large power-law network *G* with exponent $\beta >2$ is close to 1, given the appropriate scales $\Delta ,\delta$.

#### Emergent hub dynamics on power-law networks

**Fig. 6:**
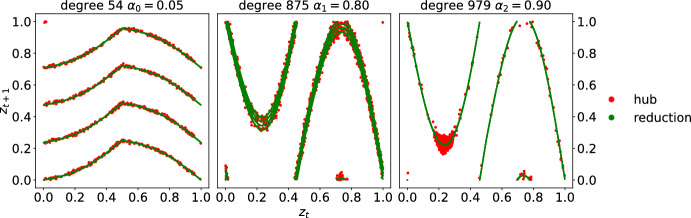
Hub dynamics of various effective coupling strengths. On power-law network $G_1$ with maximum degree $\Delta _0=979$, we run dynamics (1) at fixed coupling strength $\alpha =0.9$. The left, center, right panels concern three nodes of degree 54, 875, 979 respectively; each panel presents in red the node state $z^t$ versus next state $z^{t+1}$. The three nodes experience the mean-field dimensional reduction of effective coupling strengths $\alpha _i$ proportional to their degrees, plotted in green

Using the same isolated dynamics (2) and coupling function (3) as in Example 1.2, we fix coupling strength $\alpha =0.9$ and initialize the node states $(x_1^0,x_2^0,\cdots ,x_N^0)\in \mathbb {T}^N$ randomly uniformly in [0, 1), then iterate the $G_1$-network dynamics (1). We discard the first 5000 iterates as transients and collect the next 1000 iterates. In Figure 6 , we select three nodes of different degrees 54, 875 and 979 for the left, middle, and right panels, respectively, and plot in red the hub states $z^t$ against its next states $z^{t+1}$.

Note that the node behaviors vary drastically according to their degree. On the left panel, the dynamics of a node of degree 54 remain contractive; on the central panel, the node of degree 875 appears to have an expanding region; and lastly, on the right panel, the massive hub of degree 979 appears to hover around a deterministic fixed point near $z=0.2$. This shows the variety of node behaviors emergent from the interactions.

To explain, we continue to write $z_i^t$ for $x_i^t$ to emphasize the hubs $i\in \mathcal {H}_{\Delta }$. Split the coupling into contributions from low degree and non-low degree neighbors

$$\sum _{j=1}^N A_{ij}h_{\varvec{\omega }_i^t}(z_i^t,x_j^t)=\sum _{j\in \mathcal {N}_i\cap \mathcal {L}_{\delta }} h_{\varvec{\omega }_i^t}(z_i^t,x_j^t)+ \sum _{j\in \mathcal {N}_i\setminus \mathcal {L}_{\delta }} h_{\varvec{\omega }_i^t}(z_i^t,x_j^t);$$

the first term is the main one and sums over $\# \mathcal {N}_i\cap \mathcal {L}_{\delta }=\nu _i k_i$ contributions, whose mean can be approximated

$$ \alpha \frac{\nu _i k_i}{\Delta _0}\frac{1}{\nu _i k_i} \sum _{j\in \mathcal {N}_i\cap \mathcal {L}_{\delta }} h_{\varvec{\omega }_i^t}(z_i^t, x_j^t) \approx \alpha \frac{\nu _i k_i}{\Delta _0} \int _{\mathbb {T}} h_{\varvec{\omega }_i^t} (z_i^t,x)\textrm{d}x $$

as a space average against the Lebesgue measure. So we approximate

$$z_i^{t+1} = f_{\alpha _i, \varvec{\omega }_i^t}(z_i^t) + \xi _i^t,$$

where the reduced map $f_{\alpha ,\omega }$ was defined in eq. (5),

$$ \alpha _i:= \alpha \nu _i k_i \Delta _0^{-1} $$

is the *effective coupling strength* that hub *i* feels in the network dynamics, and the *mean-field fluctuation* of hub *i* at time *t* from initial datum $(\varvec{\omega },x)$ is, in this example,

$$\xi _i^t(\varvec{\omega },x): = \frac{\alpha _i}{\nu _ik_i} \sum _{j \in \mathcal {N}_i\cap \mathcal {L}_{\delta } }\sin 2\pi x_j^t + \frac{\alpha }{\Delta _0}\sum _{j\in \mathcal {N}_i\setminus \mathcal {L}_{\delta }} \left[ \sin 2\pi x_j^t - \sin 2\pi z_i^t - \frac{\varvec{\omega }_i^t}{3.6}\right] .$$

The first term concerns the low-degree neighbors of the hub *i*, resembles the star case and will be controlled in a similar strategy; the second term gathers all contributions from non-low degree nodes, whose dynamics are not controlled and hence will be estimated simply as $O(1-\nu _i)$ because the sum has only $(1-\nu _i)k_i$ terms, each of which is bounded.

In Figure 6, we plot in green the graph of this one-dimensional system, and the actual node behavior in red, numerically corroborating the mean-field dimensional reduction. Theorem C (i) provides the corresponding mathematical statement.

#### System size induced desynchronization

**Fig. 7:**
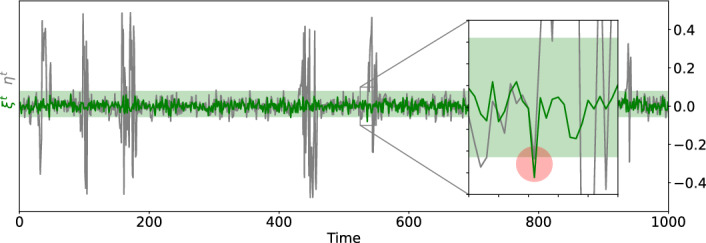
System size induced desynchronization between the two most massive hubs on a power-law network. We simulate the $G_1$-network dynamics on $N=99816$ nodes at coupling strength $\alpha =0.9$, and plot in grey the time series of hub desynchronization level $\eta ^t:= z_{i_0}^t-z_{i_1}^t$ between hubs $i_0$ of degree $\Delta _0=979$ and $i_1$ of degree 978. Large $\eta ^t$ indicate desynchronization episodes. The green time series shows comparatively small fluctuations $\xi _{i_0}^t$, with the green shaded band indicating the trapping region $[z_-,z_+]$ of the reduced dynamics $f_{0.9}$ re-centered at fixed point $z_*=f_{0.9}z_*$. The inset highlights the desynchronization mechanism, namely, an instance of a fluctuation $\xi _{i_0}^t$ sufficiently large to kick the hub $z_{i_0}^t$ out of the trapping region $[z_-,z_+]$ causes a subsequent episode of desynchronization

In Figure 7 we plot in grey the time series of the *desynchronization level*

$$ \eta ^t=z_{i_0}^t - z_{i_1}^t, $$

i.e., difference of states between the two most massively connected hubs $i_0$ of degree $\Delta _0=979$ and $i_1$ of degree 978. For this we run the $G_1$-network dynamics (1) at $\alpha =0.9$ with random initial conditions, discard the first 1500 iterated as transients, and plot for the next 1000 iterates. Here, large values of $\eta ^t$ indicate desynchronization. In fact, the simulations in [Ric16] revealed that this desynchronization becomes rare for large system size *N*. In a statistical mechanics system with continuum symmetry, similar size induced desynchronization effects have been characterized in [BGP13].

To explain this system size induced desynchronization, we note that at effective coupling strength $\alpha _i=0.9$, the reduced map $f_{0.9}=f_{0.9,\omega }$, $\omega =0,1,2,3$ for the hub *i* reads

$$f_{0.9}(z)= f_0(z) - 0.9 \sin 2\pi z.$$

It has a unique attractive fixed point $z_*\approx 0.224$, and nearby points in the trapping region $[z_-,z_+]$ are attracted towards $z_*$ uniformly. Outside this region, points may enter regions of expansion by $f_{0.9}$.

The hubs $i=i_0,i_1$ have $\alpha _i=\alpha \nu _i\kappa _i\Delta _0^{-1}$ very close to 0.9, thus remain in $[z_-,z_+]$, and syncronize with $|\eta ^t|\le z_+-z_-$, as long as $\xi _i^t$ is sufficiently small. In Figure 7 the green time series for $\xi _{i_0}^t$ ocassionally kicks $z_{i_0}^t$ out of $[z_-,z_+]$, the re-centered version $[z_--z_*, z_+ - z_*]$ shown as the green shaded band, resulting in large $\eta ^t$. The inset highlights one instance of this desynchronization mechanism.

The precise nature of the system size induced desynchronization is related to the central limit behavior of $\xi _{i}^t$ in Section 1.2.3. In fact, the fluctuations $\xi _i^t$ of any hub $i\in \mathcal {H}_{\Delta }$ satisfy similar scaling relations and Gaussian statistics as in the star network case, with the star size *L* replaced by $\nu _ik_i$, see Theorem C below. In fact, these numerical phenomena are also observed in examples beyond the setting of our Theorems, see [BRS12, Ric16].

#### Statement of main result on the power-law network

Our next results formalize the numerical observations above, namely, the locally star-like property of large power-law networks and the mean-field dimensional reduction therein with statistically controlled fluctuations.

In the limit $a\rightarrow +\infty$ or $a\rightarrow 0^+$, we use the Bachmann-Landau big-O notation $f(a)=O(g(a))$ for $\limsup |f(a)|/g(a) <+\infty$, small-o notation $f(a)=o(g(a))$ for $\lim f(a)/g(a)=0$, same-order notation $f(a) \asymp g(a)$ for the conjunction of $f(a)=O(g(a))$ and $g(a)=O(f(a))$, and asymptotic equivalence notation $f(a)\sim g(a)$ for $\lim f(a)/g(a)=1$.

##### Theorem B

**Power**-$\mathbf {Law\,Networks\,Are\,Locally\,Star}$-$\textbf{Like}$**.** Fix parameters $c_{\textrm{hub}},\lambda _{\textrm{ldn}}\in (0,1)$. Consider a large Chung-Lu network *G* generated from the power-law expected degree sequence given in (6) with *N* nodes, power-law exponent $\beta >2$, maximum expected degree $m\asymp N^{\frac{1}{\beta -1}}$, and mean expected degree $w= o(N^{\lambda _{\textrm{ldn}}\frac{1}{\beta -1}})$. By setting hub and low degree scales

$$\Delta =c_{\textrm{hub}} m,~~~~\delta \asymp \Delta ^{\lambda _{\textrm{ldn}}},$$

we regard nodes of degree above $\Delta$ as hubs and below $\delta$ as low degree. Then, with probability $1-O(N^{-1/5})$, we have

$$M\sim w^{\beta -1},~~~~L\sim N,~~~~\nu = 1 - O\left( N^{-\lambda _{\textrm{ldn}}\frac{\beta -2}{\beta -1}} w^{\beta -2}\right) ,$$

where *M* is the number of hubs, *L* the number of low degree nodes, and $\nu$ the star-like index of *G*.

**Main ideas of proof for Theorem B.** From concentration inequalities, namely, Chernoff bounds, [CL06, Lemma 5.7] can be adapted to show that the entire actual degree sequence is concentrated around the expected version. By counting, we show the locally star-like property for the expected degree sequence, and by concentration, we obtain the same for the actual degree sequence. The subtlety lies in the careful choice of the hub and low degree scales $\Delta ,\delta$ respectively. The precise statement and full proof can be found in Theorem 3.7.

##### Theorem C

$\mathbf {Hub\,Dynamics\,in\,Power}$-$\mathbf {Law\,Networks}$**.** Consider dynamics (1) on a large power-law network *G* as in Theorem B and $f_{\omega },h_{\omega }$ as in Example 1.2. Then, with initial conditions following the uniform distribution on $\mathbb {T}^N$, at coupling strength $\alpha = o\left( \min \{ N^{\lambda _{\textrm{ldn}}\frac{\beta -2}{\beta -1}} , N^{(1-\lambda _{\textrm{ldn}})/(2\beta -2)}\} \right)$, each hub $i\in \mathcal {H}_{\Delta }$ admits the mean-field dimensional reduction $f_{\alpha _i,\omega }$ at effective coupling strength $\alpha _i$ defined to be

$$\begin{aligned} f_{\alpha _i, \omega }(z):= f_{\omega }(z) + \alpha _i\int _{\mathbb {T}} h_{\omega }(z, x)\textrm{d}x\mod 1,~~~~\alpha _i:= \alpha \nu _i k_i\Delta _0^{-1}. \end{aligned}$$

More precisely, we have:

$$\mathrm {(i)}$$
**Almost sure reduction in asymptotic time:** for any
  $$\begin{aligned} \varepsilon\ge &  \max \left\{ \frac{17}{2} \alpha (1-\nu ), 34\pi \alpha ^2\delta / \Delta \right\} ,~~~~1-\nu =O\left( N^{-\lambda _{\textrm{ldn}}\frac{\beta -2}{\beta -1}} w^{\beta -2}\right) ,~~\Delta \asymp N^{\frac{1}{\beta -1}}, \\ &  \delta \asymp \Delta ^{\lambda _{\textrm{ldn}}} \asymp N^{\frac{\lambda _{\textrm{ldn}}}{\beta -1}}, \end{aligned}$$
  we have
  $$ \textrm{Prob} \left\{ (\varvec{\omega },x): \liminf _{T\rightarrow +\infty } \frac{1}{T}\sum _{t=0}^{T-1} \mathbbm {1}_{\max \{|\xi _i^t(\varvec{\omega },x)|:i\in \mathcal {H}_{\Delta }\}\le \varepsilon } \ge 1- \exp \left( -\nu \Delta \varepsilon ^2\alpha ^{-2}/19\right) \right\} =1; $$
$$\mathrm {(ii)}$$
**Small fluctuation in long time windows:** in time windows
  $$I_{t_0}^T:=\{t_0,\cdots ,t_0+T-1\},~~~~T\ge \exp (\Delta ^{1-2\kappa }),~~t_0\in \mathbb {N},$$
  we have successively small fluctuations
  $$\begin{aligned} &  \textrm{Prob} \left\{ (\varvec{\omega },x): \max _{i\in \mathcal {H}_{\Delta }} \max _{t\in I_{t_0}^T} |\xi _i^t(\varvec{\omega },x)| \le 3\alpha \Delta ^{-\kappa } \right\} \\ &  \quad \ge 1-\exp (-\Delta ^{1-2\kappa }), ~~~~0<\kappa <\min \left\{ 1/2, 1-\lambda _{\textrm{ldn}}, \lambda _{\textrm{ldn}}\frac{\beta -2}{\beta -1}\right\} ; \end{aligned}$$
$$\mathrm {(iii)}$$
**Gaussian fluctuations:** at any time $t\in \mathbb {N}$, the fluctuation $\xi _i^t$ is approximately Gaussian, i.e.,
  $$\textrm{Prob}\{(\varvec{\omega },x):\xi _i^t(\varvec{\omega },x)\le s\} \in [F_i(s-s_1) - s_2, F_i(s+s_1)+s_2],~~~~i\in \mathcal {H}_{\Delta },~~s\in \mathbb {R},$$
  where $F_i$ denotes the cdf of the normal distribution with zero mean and variance $\alpha _i^2/(2\nu _i k_i)$, and the correction constants are
  $$s_1=O(N^{-\frac{1-\lambda _{\textrm{ldn}}}{\beta -1}} + N^{-\lambda _{\textrm{ldn}} \frac{\beta -1}{\beta -2}} w^{\beta -2}),~~~~s_2=O(N^{-\frac{1}{2\beta -2}}).$$

**Main ideas of proof for Theorem C.** We combine Theorems A and B. The locally star-like property from Theorem B ensures that the hub and low degree scales are separated, allowing the low degree orbit to be shadowed by an isolated orbit; again by Theorem B, most hub neighbors are of low degree and hence well-controlled by the shadowing technique from Theorem A. When excluding the bad sets for all hubs, we use Theorem B to ensure that there are only $M\sim w^{\beta -1}$ hubs. The detailed proof of Theorem C can be found in Appendix A.2.

Our abstract result Theorem 2.18 treats the general case on a locally star-like network. In Section 3.2 the locally star-like property is verified for the random power-law network model.

We organize the rest of the paper as follows. Section 2.1 spells out all hypotheses, and develops the abstract dimensional reduction principle on the star network, which showcases the essential arguments of our shadowing plus typicality technique. Section 2.2 introduces the notion of locally star-like networks and extends the dimensional reduction technique from star to locally star-like networks. Section 3.2 constructs the random power-law network model and proves that it is locally star-like, Theorem B. Finally in Section 3 we return to the case of iid random iteration of contractions as node dynamics and complete the proof of Theorems A and C.

## Dimensional Reduction

Fix a probability preserving transformation $(\Omega ,\mathcal {F},\mathbb {P}, \theta )$ as the common model for environmental noise and a network *G* with maximum degree $\Delta _0$. On each node $i=1,,\cdots ,N$, the local dynamics are given by a random dynamical system $\varphi _i$ on the circle $\mathbb {T}=\mathbb {R}/\mathbb {Z}=[0,1]/0\sim 1$

$$\varphi _i:\mathbb {N}\times \Omega \times \mathbb {T}\rightarrow \mathbb {T},~~~~(t,\omega ,x)\mapsto \varphi _i(t,\omega ,x),$$

where $$\varphi _i$$

(i) is measurable with respect to $2^{\mathbb {N}}\otimes \mathcal {F}\otimes \mathcal {B}(\mathbb {T})$ and $\mathcal {B}(\mathbb {T})$;
(ii) satisfies the cocycle property over $\theta$, namely, $\varphi _i(0,\omega ,\cdot )=\textrm{id}_{\mathbb {T}}$ for each $\omega \in \Omega$ and $\varphi _i(t+s,\omega ,\cdot )=\varphi _i(t,\theta ^s\omega ,\cdot )\circ \varphi _i(s,\omega ,\cdot )$ for all $s,t\in \mathbb {N}$ and $\omega \in \Omega$.

Each node *i* influences its neighbor $j\in \mathcal {N}_i$ with contribution $h(\omega ,x_j,x_i)$ given by a random pairwise coupling function $h:\Omega \times \mathbb {T}^2\rightarrow \mathbb {R}$. At coupling strength $\alpha >0$, the network dynamical system is the RDS

7 $$\begin{aligned} \Phi _{\alpha }:\mathbb {N}\times \Omega \times \mathbb {T}^N\rightarrow \mathbb {T}^N, \end{aligned}$$

where each node $(\Phi _{\alpha }(t,\omega ,x))_i=x_i^t$ evolves by

8 $$\begin{aligned} x_i^{t+1} = \varphi _i(1,\theta ^t\omega ,x_i^t) + \alpha \cdot \frac{1}{\Delta _0} \sum _{j=1}^N A_{ij}h(\theta ^t\omega ,x_i^t,x_j^t)\mod 1,~~~~i=1,\cdots ,N, \end{aligned}$$

where $A=(A_{ij})_{i,j}$ is the same adjacency matrix of network *G* as in Eq. (1). Note that the trajectory $\{x_j^t:t\in \mathbb {N}\}$ of low degree node $j\in \mathcal {L}_{\delta }$ is a pseudo-orbit of $\varphi _j$ with error at each time step bounded by $\alpha \Delta _0^{-1} \delta \Vert h(\theta ^t\omega ,\cdot ,\cdot )\Vert _{C^0}$.

We assume the following conditions on the random dynamical system (7):

1. Independent and identically distributed node maps: the random variables $\omega \mapsto \varphi _i(1,\theta ^t\omega ,\cdot )$, $i=1,\cdots ,N$, $t\in \mathbb {N}$ take values in the space of continuous circle maps, have the same distribution, and are independent in time *t* and node *i*;
2. Unique stationary measure of node dynamics: the isolated node dynamics $\varphi _i$, $i=1,\cdots ,N$ admits a unique stationary measure *m*;
3. $C^4$ pairwise coupling maps: $h(\theta ^t\omega ,\cdot ,\cdot )$, $t\in \mathbb {N}$ share the same distribution in the space $C^4(\mathbb {T}^2;\mathbb {R})$ of $C^4$-smooth maps $\mathbb {T}^2\rightarrow \mathbb {R}$ and are independent in time *t*.

### Remark 2.1

In fact, we may allow in (R1–2) that the low-degree node maps $\varphi _j$ share the same map distribution with unique stationary measure *m*, while the other node maps enjoy different distributions. The smoothness $C^4$ in (R3) is assumed to ensure sufficient decay of the Fourier coefficients. In (R3) we may also allow the coupling maps $h_i(\omega ,x_i,x_j)$ to vary among nodes *i*, as long as all neighbors $j\in \mathcal {N}_i$ of any node *i* influence it via the same coupling map $h_i$. Under assumptions (R1–3) the network dynamical system $\Phi _{\alpha }$ is an iid random iteration of continuous maps $\Phi _{\alpha }(1,\theta ^t\omega ,\cdot )$ on $\mathbb {T}^{N}$.

### Remark 2.2

*(Notation of* $\omega$*)*. To avoid notational cluter, we have changed the notation of noise. Now we only use the simple font $\omega$ as an element of the abstract probability space $\Omega$ to denote noise realization. The fact that the noise is iid in time $t=0,1,\cdots$ and node coordinates $i=1,\cdots ,N$ is reflected in node dynamics $\varphi _i(t,\omega ,\cdot )$ and coupling function $h(\theta ^t\omega ,\cdot ,\cdot )$.

### Reduction on the star network

When *G* is a star graph consisting of 1 hub and $L=N-1$ low degree nodes, the hub evolves by

9 $$\begin{aligned} z^{t+1}= \varphi _1(1,\theta ^t\omega ,z^t)+ \alpha \cdot \frac{1}{L}\sum _{j=2}^N h(\theta ^t \omega ,z^t,x_j^t)\mod 1, \end{aligned}$$

and each low-degree node evolves by

10 $$\begin{aligned} x_j^{t+1} = \varphi _j(1,\theta ^t\omega ,x_j^t) + \alpha \cdot \frac{1}{L} h(\theta ^t\omega ,x_j^t,z^t) \mod 1,~~~~j=2,\cdots ,N. \end{aligned}$$

The mean-field reduction seeks to approximate the mass action of the *L* low-degree nodes on the hub *z* by the space average against *m* on $\mathbb {T}$

$$\alpha \frac{1}{L} \sum _{j=2}^N h(\omega ,z,x_j)= \alpha \int _{\mathbb {T}} h(\omega ,z,y)\textrm{d} m(y) + \xi _m(\omega ,x),$$

where the corresponding fluctuation $\xi _m(\omega ,x)$ is given by

11 $$\begin{aligned} \alpha ^{-1}\xi _m(\omega ,x)= \frac{1}{L} \sum _{j=2}^N h(\omega ,z,x_j) - \int _{\mathbb {T}} h(\omega ,z,y)\textrm{d}m(y). \end{aligned}$$

This way, the hub behavior in the *N*-dimensional network dynamics on the undirected star becomes reduced to (approximated by) the one-dimensional random system $\varphi _{\alpha ,m}:\mathbb {N}\times \Omega \times \mathbb {T}\rightarrow \mathbb {T}$, recursively defined by

12 $$\begin{aligned} \varphi _{\alpha ,m}(t+1,\omega ,z)=\varphi _1(1,\theta ^t\omega ,\varphi _{\alpha ,m}(t,\omega ,z)) +\alpha \int _{\mathbb {T}} h(\theta ^t\omega ,\varphi _{\alpha ,m}(t,\omega ,z),y)\textrm{d} m(y). \end{aligned}$$

In this notation, the hub evolution becomes

$$z^{t+1}= \varphi _{\alpha ,m}(1,\theta ^t\omega ,z^t) + \xi _m(\theta ^t\omega ,x^t).$$

#### Definition 2.3

We say that the *hub* (9) *admits* $\varepsilon$-*reducion to* $\varphi _{\alpha ,m}$ *on initial data* $(\omega ,x)\in \Omega \times \mathbb {T}^N$ *at time* *t* if

$$| \xi _m(\theta ^t\omega ,\Phi _{\alpha }(t,\omega ,x))|\le \varepsilon ,$$

and that it admits $\varepsilon$-reduction to $\varphi _{\alpha ,m}$ on initial data $(\omega ,x)\in \Omega \times \mathbb {T}^N$ with exceptional frequency at most $\rho$ if

$$ \limsup _{T\rightarrow +\infty } \frac{1}{T} \# \left\{ t\in [0,T-1]: | \xi _m(\theta ^t\omega ,\Phi _{\alpha }(t,\omega ,x))|>\varepsilon \right\} \le \rho . $$

#### Theorem 2.4

(Reduction theorem on a star) Suppose (R1–3) hold for the star network random dynamical system (9-10) on $N-1$ low-degree nodes and one hub node at coupling strength $\alpha >0$. Let initial data $(\omega ,x)\in \Omega \times \mathbb {T}^{N}$ be such that the network trajectory $\{x^t=\Phi _{\alpha }(t,\omega ,x): t\in \mathbb {N}\}$ admits an *m*-typical shadowing orbit in the low degree coordinates; more precisely, there is $(\omega _{{s}},x_{{s}})\in \Omega \times \mathbb {T}^{N}$ satisfying

Shadowing $$\begin{aligned} \sup _{t\in \mathbb {N}}\max _{j=2,\cdots ,N} d_{\mathbb {T}}(x_j^t, \varphi _j(t,\omega _{{s}},x_{{s},j}))\le \varepsilon _s \left( \alpha (N-1)^{-1} \sup _{t\in \mathbb {N}} \Vert h(\theta ^t\omega ,\cdot ,\cdot )\Vert _{C^0}\right) ; \end{aligned}$$

Typicality $$\begin{aligned} \frac{1}{T}\sum _{t=0}^{T-1} \delta _{\varphi _2(t,\omega _{{s}},x_{{s},2})} \otimes \cdots \otimes \delta _{\varphi _N(t,\omega _{{s}},x_{{s},N})}\xrightarrow [T\rightarrow +\infty ]{\text {weak}^*} m^{\otimes (N-1)}, \end{aligned}$$

where $d_{\mathbb {T}}$ denotes the distance on the circle, the shadowing precision $\delta _s\mapsto \varepsilon _s (\delta _s )$ is a $\mathbb {R}_+$-valued function converging to 0 as $\delta _s$ tends to 0. Then, for any error tolerance

$$\varepsilon \ge \max \left\{ \alpha (N-1)^{-1/2},4\alpha \sup _{t\in \mathbb {N}}|h(\theta ^t \omega ,\cdot ,\cdot )|_{\textrm{Lip}} \varepsilon _s \left( \alpha (N-1)^{-1} \sup _{t\in \mathbb {N}} \Vert h(\theta ^t\omega ,\cdot ,\cdot )\Vert _{C^0} \right) \right\} ,$$

the hub behavior (9) admits $\varepsilon$-reducion to $\varphi _{\alpha ,m}$ on initial data $(\omega ,x)\in \Omega \times \mathbb {T}^N$ with exceptional asymptotic frequency at most $\rho$ with

$$\begin{aligned} &  \rho (\varepsilon ,\omega )=D(\varepsilon ,\omega ) \exp (-(N-1)\varepsilon ^2 \alpha ^{-2} c(\omega )),~~~~D(\varepsilon ,\omega )\asymp \varepsilon ^{-1} \sup _{t\in \mathbb {N}}\Vert h(\theta ^t\omega ,\cdot ,\cdot )\Vert _{C^4},\\ &  \quad c(\omega )=\frac{450}{\sup _{t\in \mathbb {N}} \Vert h(\theta ^t\omega ,\cdot ,\cdot )\Vert _{C^4}}, \end{aligned}$$

where the constants $D(\varepsilon ,\omega )$ and $c(\omega )$ are independent of *N* and *x*.

#### Remark 2.5

(Typicality) condition assumes that the shadowing orbit $(\varphi _2(t,\omega _{{s}},x_{{s},2}),\cdots ,\varphi _N(t,\omega _{{s}},x_{{s},N}))$ is $m^{\otimes L}$-typical, $L=N-1$, i.e., follows the distribution $m^{\otimes L}$ given by the unique stationary meausre. Note that Theorem 2.4 (Shadowing) plus (Typicality) for one initial data $(\omega ,x)\in \Omega \times \mathbb {T}^{N}$ and concludes the reduction for this particular instance of initial data. To obtain Shadowing and Typicality conditions for $\mathbb {P}\otimes m^{\otimes N}$-a.e. $(\omega ,x)\in \Omega \times \mathbb {T}^N$ as in Theorem A, one tends to encounter the following scenario. By local hyperbolicity of node dynamics $\varphi _i$, the shadowing technique produces some initial datum $(\omega _s,x_s)$, whose random orbit $\varphi _i(t,\omega _s,x_s)$ achieves (Shadowing). And we wish to establish (Typicality) for $\varphi _i(t,\omega _s,x_s)$. For this, the existence and uniqueness of stationary measure *m* from (R2) ensures, see [BW22, Theorem 16.4], the ergodicity of the Markov chain associated to the iid random iteration $\varphi _i$ on each node $i=1,\cdots ,N$ with initial distribution *m*.

1. By Breiman’s ergodic theorem [Bre60], for each $x_i\in \mathbb {T}$, there is $\Omega _{x_i}\subseteq \Omega$ with $\mathbb {P}(\Omega _{x_i})=1$ such that the random orbit $\varphi _i(t,\omega ,x_i)$ is *m*-typical for every $\omega \in \Omega _{x_i}$. However, this typicality falls short because there may be pathological situations where the shadowing initial datum $(\omega _s,x_{s,i})$ is such that $\omega _s \notin \Omega _{x_{s,i}}$. This is the case with random expanding or hyperbolic maps as node dynamics.
2. For random *uniform contractions*, such as those in Example 1.2, Theorem 3.3 establishes a uniform $\Omega _*$ with $\mathbb {P}(\Omega _*)=1$ such that the random orbit $\varphi _i(t,\omega ,x_i)$ is *m*-typical for every $(\omega ,x_i)\in \Omega _*\times \mathbb {T}$. Moreover, we show that $(\omega _s,x_{s})=(\omega ,x)$, see Corollary 3.5. Thus, Shadowing and Typicality conditions are achieved for every $(\omega ,x)\in \Omega _*\times \mathbb {T}^N$.

#### Remark 2.6

If $\mathbb {P}\otimes m^{\otimes N}$-a.e. $(\omega ,x)\in \Omega \times \mathbb {T}^N$ admits shadowing intial data $(\omega _{{s}},x_{{s}})$ satisfying (Shadowing) and (Typicality), then the $\varepsilon$-reduction holds $\mathbb {P}\otimes m^{\otimes N}$-almost surely by Theorem 2.4. In the case of Example 1.2, we have $(\omega ,x)= (\omega _{{s}},x_{{s}})$ and hence obtain (ii) Small fluctuation in long time windows and (iii) Gaussian fluctuations, as in Theorem A. See Appendix A for more details.

#### Remark 2.7

As in the usual Shadowing Lemmas, $\epsilon _s(\delta _s)$ in Theorem 2.4 is the shadowing precision which upper bounds the distance between the shadowing and pseudo orbits; it is a function of the error tolerance $\delta _s$ of the pseudo orbit. In Example 1.2, we have $\delta _s=\alpha L^{-1}$ and $\epsilon _s(\delta _s) = \frac{\delta _s}{1-\lambda }= \frac{\alpha L^{-1}}{1-\lambda }$, where $\lambda =1/2$ is the contraction rate, see also Corollary 3.5. Our result relies on the hyperbolicity of the low degree node dynamics alone in terms of the shadowing property, which allows us to overcome the shortcoming i) of [PvST20] as discussed in Introduction.

#### Decay of Fourier coefficients

We start with some preparations in Fourier analysis. Write the Fourier series of the coupling maps $h(\omega ,\cdot ,\cdot )\in C^4(\mathbb {T}^2,\mathbb {R})$

$$h(\omega ,x_1,x_2)= \sum _{(n_1,n_2)\in \mathbb {Z}^2}a^{\omega }_{(n_1,n_2)} e^{2\pi i(n_1x_1+n_2x_2)},$$

where the Fourier coefficients are defined by

$$\begin{aligned} a^{\omega }_{(n_1,n_2)}&:= \int _0^1 \int _0^1 e^{-2\pi i(n_1x_1+n_2x_2)} h(\omega ,x_1,x_2)\textrm{d}x_1\textrm{d}x_2. \end{aligned}$$

We gather some basic facts of decay of Fourier coefficients for smooth functions from [Gra14, Theorem 3.3.9].

##### Lemma 2.8

(Decay of Fourier coefficients). For $h(\omega ,\cdot ,\cdot )\in C^4(\mathbb {T}^2;\mathbb {R})$, $(n_1,n_2)\in \mathbb {Z}^2$ and multi-index $(m_1,m_2)\in \mathbb {N}^2$ with $\sum _{j=1}^2 m_j\le 4$, we have

$$ (2\pi )^{m_1+m_2}|n_1|^{m_1} |n_2|^{m_2} |a^{\omega }_{(n_1,n_2)}|\le \Vert h(\omega ,\cdot ,\cdot )\Vert _{C^4}.$$

In particular, we obtain

$$\begin{aligned} |a^{\omega }_{(0,0)}|&\le \Vert h(\omega ,\cdot ,\cdot )\Vert _{C^4};\\ |a^{\omega }_{(0,n_2)}|&\le \frac{1}{(2\pi )^4 n_2^4} \Vert h(\omega ,\cdot ,\cdot )\Vert _{C^4},~~~~\forall n_2\ne 0;\\ |a^{\omega }_{(n_1,0)}|&\le \frac{1}{(2\pi )^4 n_1^4} \Vert h(\omega ,\cdot ,\cdot )\Vert _{C^4},~~~~\forall n_1\ne 0;\\ |a^{\omega }_{(n_1,n_2)}|&\le \frac{1}{(2\pi )^4 n_1^2 n_2^2}\Vert h(\omega ,\cdot ,\cdot )\Vert _{C^4},~~~~\forall n_1\ne 0\text { and }n_2\ne 0, \end{aligned}$$

so that its Fourier series

$$\begin{aligned} h(\omega ,x_1,x_2)&= \sum _{(n_1,n_2)\in \mathbb {Z}^2} a^{\omega }_{(n_1,n_2)} e^{2\pi i (n_1x_1+n_2x_2)} = a^{\omega }_{(0,0)} + \sum _{n_2\ne 0} a^{\omega }_{(0,n_2)} e^{2\pi i n_2x_2} \\&\quad + \sum _{n_1\ne 0} a^{\omega }_{(n_1,0)} e^{2\pi i n_1x_1} \\&\quad + \sum _{n_1\ne 0 \text { and } n_2\ne 0} a^{\omega }_{(n_1,n_2)} e^{2\pi i (n_1x_1+n_2x_2)} \end{aligned}$$

converges absolutely and uniformly.

#### Bad sets

For $\phi \in C^0(\mathbb {T};\mathbb {R})$, we define its bad set to be

$$B(\varepsilon ,\phi ):=\left\{ {x}\in \mathbb {T}^N: \left| \frac{1}{L} \sum _{j=2}^N \phi ({x}_j)- \int _{\mathbb {T}} \phi \textrm{d}m \right| >\varepsilon \right\} .$$

In the Introduction example, the bad set $B(\varepsilon ,\phi )$ with $\phi (x)=\sin 2\pi x$ is precisely the part of state space that produces large fluctuation $|\xi ^t|>\varepsilon$. As we will see shortly, the bad sets play a similar role in the general case and thus good control on the frequency of visits to them is key to obtaining $\varepsilon$-reduction. We first estimate the size of the bad set and frequency of visit to it by trajectories of the product system.

##### Proposition 2.9

(Size of Bad Set) The bad set $B(\varepsilon ,\phi )$ has exponentially small $m^{\otimes N}$-volume:

$$m^{\otimes N} (B(\varepsilon ,\phi ))\le 2\exp (-L2^{-1}\Vert \phi \Vert _{\infty }^{-2} \varepsilon ^2).$$

##### Proof

For $i=2,\cdots , N$, define random variable $X_i$ on $(\mathbb {T}^N, m^{\otimes N})$ by

$$ X_i:\mathbb {T}^N\rightarrow \mathbb {T},~~~~(x_1,\cdots ,x_N)\mapsto \phi (x_i).$$

Then, these random variables $X_2\cdots ,X_N$ are independent and bounded. By Hoeffding’s Inequality [Ver18, Theorem 2.2.2], for any $\varepsilon >0$, we have

$$m^{\otimes N} (B(\varepsilon ,\phi ))\le 2\exp (-L2^{-1}\Vert \phi \Vert _{\infty }^{-2} \varepsilon ^2),$$

as desired. $$\square $$

##### Remark 2.10

Note that the geometric structure of the bad set $B(\varepsilon ,\phi )$ may be complicated, depending on the choice of $\phi$. However, its $m^{\otimes L}$-volume has an exponentially small upper bound, regardless of $\phi$.

##### Lemma 2.11

(Random Orbit Visits Bad Set with Small Frequency). Under the hypotheses of Reduction Theorem 2.4, the uncoupled orbit of the shadowing pair $(\omega _{{s}},x_{{s}})\in \Omega \times \mathbb {T}^{N}$ visits the bad set with exponentially small frequency:

$$\limsup _{T\rightarrow +\infty } \frac{1}{T}\sum _{t=0}^{T-1} \mathbbm {1}_{B(\varepsilon ,\phi )} (\varphi _2(t,\omega _{{s}},x_{{s},2}),\cdots ,\varphi _N(t,\omega _{{s}},x_{{s},N}) )\le 2\exp (-L2^{-1}\Vert \phi \Vert _{\infty }^{-2} \varepsilon ^2).$$

##### Proof

We emphasize only the dependence on $\varepsilon$ and suppress other dependencies by writing $B_{\varepsilon }$ for $B(\varepsilon ,\phi )$. First we treat the case where the bad set has null boundary:

$$m^{\otimes L}(\partial B_{\varepsilon })=0.$$

From the $\hbox {weak}^*$ convergence in (Typicality) assumption in Theorem 2.4, we conclude the frequency of visit by Portmanteau Theorem and Proposition 2.9

$$\lim _{T\rightarrow +\infty } \frac{1}{T}\sum _{t=0}^{T-1} \mathbbm {1}_{B_{\varepsilon }} (\varphi _2(t,\omega _{s},x_{{s},2}),\cdots ,\varphi _N(t,\omega _{s},x_{{s},N}) ) = m^{\otimes L}(B_{\varepsilon })\le 2\exp (-L2^{-1}\Vert \phi \Vert _{\infty }^{-2} \varepsilon ^2).$$

Note that the bad set is the superlevel set of the continuous function $\mathbb {T}^L\rightarrow \mathbb {R}$, $x\mapsto \left| \frac{1}{L}\sum _{j=2}^N \phi (x_j) - \int _{\mathbb {T}}\phi \textrm{d}m\right|$, and hence $B_{\varepsilon }= \bigcup _{\varepsilon '\in [0,\varepsilon )} \partial B_{\varepsilon '}$ is a disjoint union. Since $m^{\otimes L}(B_{\varepsilon })<\infty$, it follows that $m^{\otimes L}(\partial B_{\varepsilon '})>0$ for at most countably many $\varepsilon '\in [0,\varepsilon )$. Hence, there are $\varepsilon '\in (0,\varepsilon )$ arbitrarily close to $\varepsilon$ with $m^{\otimes L}(\partial B_{\varepsilon '})=0$.

Fix any such $\varepsilon '\in (0, \varepsilon )$. We then have $B_{\varepsilon }\subseteq B_{\varepsilon '}$ and $\mathbbm {1}_{B_{\varepsilon }}\le \mathbbm {1}_{B_{\varepsilon '}}$. Therefore, from the special case of null boundary, we obtain

$$\begin{aligned}&\limsup _{T\rightarrow +\infty } \frac{1}{T}\sum _{t=0}^{T-1} \mathbbm {1}_{B_{\varepsilon }} (\varphi _2(t,\omega _{{s}},x_{{s},2}),\cdots ,\varphi _N(t,\omega _{{s}},x_{{s},N}) ) \\&\quad \le \limsup _{T\rightarrow +\infty } \frac{1}{T}\sum _{t=0}^{T-1} \mathbbm {1}_{B_{\varepsilon '}}(\varphi _2(t,\omega _{{s}},x_{{s},2}),\cdots ,\varphi _N(t,\omega _{{s}},x_{{s},N}) )= m^{\otimes L}(B_{\varepsilon '})\\&\quad \le 2\exp (-L2^{-1}\Vert \phi \Vert _{\infty }^{-2} (\varepsilon ')^2). \end{aligned}$$

But since $\varepsilon '\in (0,\varepsilon )$ can be chosen arbitrarily close to $\varepsilon$, the estimates follow. $\square$

When dealing with multiple bad sets $B_1,\cdots ,B_D$, each with asymptotic frequency at most $\rho _k$ of the trajectory visiting $B_k$, $k=1,\cdots ,D$, we can lower bound the asymptotic frequency of the trajectory visiting none of the bad sets by $1- \sum _{k=1}^D \rho _k$.

#### Proof of reduction theorem on a star

We will split the fluctuation estimates

$$\begin{aligned} \xi _{m}(\theta ^t\omega ,x^t)&= \alpha \frac{1}{L} \sum _{j=2}^N h(\theta ^t\omega ,z^t,x_j^t) - \alpha \int _{\mathbb {T}} h(\theta ^t\omega ,z^t,y)\textrm{d}m(y) \end{aligned}$$

into three parts. By (Shadowing), the first term

decoupling $$\begin{aligned} \zeta _d(t,\omega ,x,\omega _{{s}}, x_{{s}})&:= \alpha \frac{1}{L} \sum _{j=2}^N \left[ h(\theta ^t\omega , z^t,x_j^t) - h(\theta ^t\omega ,z^t,x_{{s},j}^t) \right] , \end{aligned}$$

decouples the low degree node orbit $x_j^t=\Phi _{\alpha }(t,\omega ,x)_j$ by comparing it with the isolated shadowing orbit $x_{{s},j}^t = \varphi _j(t,\omega _{{s}}, x_{{s},j})$, which will be controlled by the shadowing precision and Lipschitz continuity of $h(\theta ^t\omega ,\cdot ,\cdot )$, see Lemma 2.12. We now have

$$\xi _{m}(\theta ^t\omega ,x^t)= \zeta _d(t,\omega ,x,\omega _{{s}},x_{{s}})+ \alpha \left[ \frac{1}{L} \sum _{j=2}^N h(\theta ^t\omega ,z^t,x_{{s},j}^t) - \int _{\mathbb {T}} h(\theta ^t\omega ,z^t,y)\textrm{d}m(y)\right] .$$

By (Typicality), the shadowing orbit $\left( x_{{s},2}^t,\cdots ,x_{{s},N}^t\right)$ distributes in time as $m^{\otimes L}$ and thus visits any bad set with controlled asymptotic frequency. In order to find the appropriate bad sets, we take Fourier series expansion

$$ h(\theta ^t\omega ,x_1,x_2) = \sum _{(n_1,n_2)\in \mathbb {Z}^2} a^{\theta ^t\omega }_{(n_1,n_2)} \phi _{n_1}(x_1)\phi _{n_2}(x_2), $$

where $\phi _n(x):=e^{2\pi i nx}$ and note that the Fourier modes corresponding to $n_2=0$ drop out

$$a_{(n_1,0)}^{\theta ^t\omega } \phi _{n_1}(z^t)\left[ \phi _0(x_{{s},j}) - \int _{\mathbb {T}} \phi _0(y)\textrm{d}m(y)\right] =0.$$

The high Fourier modes $\phi _{n_2}$ with $|n_2|>D$ for a suitable cutoff level *D*, make up the second term

HFM $$\begin{aligned} \zeta _h (t,\omega ,x,\omega _{{s}}, x_{{s}})&:= \alpha \sum _{|n_2|> D} \left[ a_{(0,n_2)}^{\theta ^t\omega } + \sum _{n_1\ne 0} a_{(n_1,n_2)}^{\theta ^t\omega } \phi _{n_1}(z^t) \right] \\&\quad \left[ \frac{1}{L} \sum _{j=2}^N\phi _{n_2}(x_{{s},j}^t) - \int _{\mathbb {T}} \phi _{n_2}(y) \textrm{d}m(y)\right] , \end{aligned}$$

which will be controlled by decay of Fourier coefficients, see Lemma 2.13.. The low Fourier modes $\phi _{n_2}$ with $1\le |n_2|\le D$ make up the third term

LFM $$\begin{aligned} \zeta _{\ell } (t,\omega ,x,\omega _{{s}}, x_{{s}})&:= \alpha \sum _{1\le |n_2|\le D} \left[ a_{(0,n_2)}^{\theta ^t\omega } + \sum _{n_1\ne 0} a_{(n_1,n_2)}^{\theta ^t\omega } \phi _{n_1}(z^t) \right] \\&\quad \left[ \frac{1}{L} \sum _{j=2}^N\phi _{n_2}(x_{{s},j}^t) - \int _{\mathbb {T}} \phi _{n_2}(y) \textrm{d}m(y)\right] , \end{aligned}$$

which will be controlled by rare visits of the shadowing orbit $\left( x_{{s},2}^t,\cdots ,x_{{s},N}^t\right)$ to the bad sets $B(\varepsilon _b,\phi _{n_2}), 1\le |n_2|\le D$, see Lemma 2.14. In the next three lemmas, we estimate each of $\zeta _d,\zeta _h,\zeta _{\ell }$.

##### Lemma 2.12

(Shadowing estimates). Under the hypotheses of Theorem 2.4, we control the $\mathrm {(decoupling)}$ term

$$ \left| \zeta _d(t,\omega ,x,\omega _{{s}}, x_{{s}}) \right| \le \varepsilon /4,~~~~t\in \mathbb {N}. $$

##### Proof of Lemma 2.12

By Lipschitz continuity of $h(\omega ,\cdot ,\cdot )\in C^4(\mathbb {T}^2,\mathbb {R})$, we have

$$\begin{aligned} \left| \zeta _d (t,\omega ,x,\omega _{{s}}, x_{{s}}) \right|&\le \alpha \frac{1}{L} L |h(\theta ^t\omega ,z^t,\cdot )|_{\textrm{Lip}} d_{\mathbb {T}}(x_j^t, x_{{s},j}^t) \\&\le \alpha \sup _{t\in \mathbb {N}} |h(\theta ^t\omega ,\cdot ,\cdot )|_{\textrm{Lip}} \varepsilon _s (\alpha L^{-1} \sup _{t\in \mathbb {N}} \Vert h(\theta ^t\omega ,\cdot ,\cdot )\Vert _{C^0}). \end{aligned}$$

The estimate follows by the assumption $\varepsilon \ge 4\alpha \sup _{t\in \mathbb {N}}|h(\theta ^t\omega ,\cdot ,\cdot )|_{\textrm{Lip}} \varepsilon _s(\alpha L^{-1} \sup _{t\in \mathbb {N}} \Vert h(\theta ^t\omega ,\cdot ,\cdot )\Vert _{C^0}).$ $\square$

##### Lemma 2.13

(High Fourier modes controlled by decay of Fourier coefficients). Under the hypotheses of Theorem 2.4, by choosing $D(\varepsilon ,\omega ) \asymp \varepsilon ^{-1}\sup _{t\in \mathbb {N}}\Vert h(\theta ^t\omega ,\cdot ,\cdot )\Vert _{C^4}$, we estimate $\mathrm {(HFM)}$

$$ \left| \zeta _h (t,\omega ,x,\omega _{{s}}, x_{{s}})\right| \le \varepsilon /2,~~~~\forall t\in \mathbb {N}. $$

##### Proof of Lemma 2.13

By decay of Fourier coefficients Lemma 2.8, we estimate (HFM)

$$\begin{aligned} \left| \zeta _h(t,\omega ,x,\omega _{{s}}, x_{{s}})\right|&\le \alpha \sum _{|n_2|>D} \frac{\Vert h(\theta ^t\omega ,\cdot ,\cdot )\Vert _{C^4}}{ (2\pi )^4 n_2^4} \frac{1}{L} L2\Vert \phi _{n_2}\Vert _{\infty } \\&\quad + \alpha \sum _{n_1\ne 0}\sum _{|n_2|>D} \frac{\Vert h(\theta ^t\omega ,\cdot ,\cdot )\Vert _{C^4}}{(2\pi )^4 n_1^2 n_2^2} \frac{1}{L} L 2 \Vert \phi _{n_2}\Vert _{\infty } \\&\le \frac{2\alpha \sup _{t\in \mathbb {N}}\Vert h(\theta ^t\omega ,\cdot ,\cdot )\Vert _{C^4}}{ (2\pi )^4}\sum _{|n_2|>D} 1/n_2^4 + \frac{2\alpha \sup _{t\in \mathbb {N}} \Vert h(\theta ^t\omega ,\cdot ,\cdot )\Vert _{C^4}}{(2\pi )^4}\\&\quad \sum _{n_1\ne 0}\frac{1}{n_1^2} \sum _{|n_2|>D}\frac{1}{n_2^2} \\&= \frac{2\alpha \sup _{t\in \mathbb {N}}\Vert h(\theta ^t\omega ,\cdot ,\cdot )\Vert _{C^4}}{ (2\pi )^4}\sum _{|n_2|>D} 1/n_2^4 + \frac{\alpha \sup _{t\in \mathbb {N}} \Vert h(\theta ^t\omega ,\cdot ,\cdot )\Vert _{C^4}}{24\pi ^2} \\&\quad \sum _{|n_2|>D}\frac{1}{n_2^2}, \end{aligned}$$

where we have used the identity $\sum _{n=1}^{\infty }1/n^2=\pi ^2/6$ in the last equality. By choosing $D\ge \max \{D_1(\varepsilon ,\omega ),D_2(\varepsilon ,\omega )\}$, where $D_1(\varepsilon ,\omega )$ is so large that

$$\frac{2\alpha \sup _{t\in \mathbb {N}}\Vert h(\theta ^t\omega ,\cdot ,\cdot )\Vert _{C^4}}{ (2\pi )^4} \sum _{|n|>D_1(\varepsilon ,\omega )}1/n^4\le \varepsilon /4,$$

and $D_2(\varepsilon ,\omega )$ is so large that

$$\frac{\alpha \sup _{t\in \mathbb {N}}\Vert h(\theta ^t\omega ,\cdot ,\cdot )\Vert _{C^4}}{ 24\pi ^2}\left( \sum _{|n|>D_2(\varepsilon ,\omega )}1/n^2\right) \le \varepsilon /4,$$

we obtain that $\left| \zeta _h(t,\omega ,x,\omega _{{s}}, x_{{s}})\right| \le \varepsilon /2.$ Since $\sum _{|n|>D} 1/n^4 \asymp D^{-3}$, we take $D_1(\varepsilon ,\omega )\asymp \varepsilon ^{-1/3}\sup _{t\in \mathbb {N}}\Vert h(\theta ^t\omega ,\cdot ,\cdot )\Vert _{C^4}$; since $\sum _{|n|>D} 1/n^2 \asymp D^{-1}$, we take $D_2(\varepsilon ,\omega )\asymp \varepsilon ^{-1}\sup _{t\in \mathbb {N}}\Vert h(\theta ^t\omega ,\cdot ,\cdot )\Vert _{C^4}$. $\square$

##### Lemma 2.14

(Low Fourier modes controlled by rare visits to bad sets). Under the hypotheses of Theorem 2.4, by choosing $D(\varepsilon ,\omega ) \asymp \varepsilon ^{-1} \sup _{t\in \mathbb {N}}\Vert h(\theta ^t\omega ,\cdot ,\cdot )\Vert _{C^4}$, we control $\mathrm {(LFM)}$

$$ \left| \zeta _{\ell } (t,\omega ,x,\omega _{{s}}, x_{{s}}) \right| \le \varepsilon /4 $$

with exponentially small exceptional asymptotic frequency at most $\rho$ with

$$\rho (\varepsilon ,\omega )= 4D (\varepsilon ,\omega ) \exp \left( -L \frac{450\varepsilon ^2}{\alpha ^2\sup _{t\in \mathbb {N}}\Vert h(\theta ^t\omega ,\cdot ,\cdot )\Vert ^2_{C^4}}\right) .$$

##### Proof of Lemma 2.14

We estimate $\zeta _{\ell } (t,\omega ,x,\omega _{{s}}, x_{{s}})$ as a problem of frequency of visits to the bad sets $B_n:= B(\varepsilon _b, \phi _n)$, $n=\pm 1,\cdots ,\pm D$. For each such *n*, Lemma 2.11 implies that the shadowing orbit $x_{{s}}^t = (\varphi _2(t,\omega _{{s}}, x_{{s},2}),\cdots ,\varphi _N(t,\omega _{{s}}, x_{{s},N}))$ visits $B_n$ with exponentially small frequency at most $2\exp (-L 2^{-1} \Vert \phi _n\Vert _{\infty }^{-2}\varepsilon _b^2)=2\exp (-L 2^{-1} \varepsilon _b^2)$. Whenever all such bad sets are avoided, we can bound (LFM)

$$\begin{aligned} \left| \zeta _{\ell } (t,\omega ,x,\omega _{{s}}, x_{{s}}) \right|&\le \alpha \sum _{1\le |n_2|\le D} \left[ |a_{(0,n_2)}^{\theta ^t\omega }| + \sum _{n_1\ne 0} |a_{(n_1,n_2)}^{\theta ^t\omega }| \right] \\&\quad \left| \frac{1}{L} \sum _{j=2}^N\phi _{n_2}(x_{{s},j}^t) - \int _{\mathbb {T}} \phi _{n_2}(y) \textrm{d}m(y)\right| \\&\le \alpha \sum _{1\le |n_2|\le D} \left[ \frac{\sup _{t\in \mathbb {N}}\Vert h(\theta ^t\omega ,\cdot ,\cdot )\Vert _{C^4}}{(2\pi )^4 n_2^4} + \sum _{n_1\ne 0} \frac{\sup _{t\in \mathbb {N}}\Vert h(\theta ^t\omega ,\cdot ,\cdot )\Vert _{C^4}}{(2\pi )^4 n_1^2n_2^2} \right] \varepsilon _b\\&\le \frac{\alpha \sup _{t\in \mathbb {N}}\Vert h(\theta ^t\omega ,\cdot ,\cdot )\Vert _{C^4}}{(2\pi )^4 } \left[ \frac{2\pi ^4}{90} + \frac{2\pi ^2}{6}\frac{2\pi ^2}{6} \right] \varepsilon _b \\&= \frac{\alpha \sup _{t\in \mathbb {N}}\Vert h(\theta ^t\omega ,\cdot ,\cdot )\Vert _{C^4}}{120 } \varepsilon _b. \end{aligned}$$

By choosing

13 $$\begin{aligned} \varepsilon _b=\frac{30}{\alpha \sup _{t\in \mathbb {N}}\Vert h(\theta ^t\omega ,\cdot ,\cdot )\Vert _{C^4}}\varepsilon , \end{aligned}$$

we obtain $\left| \zeta _{\ell } (t,\omega ,x,\omega _{{s}}, x_{{s}}) \right| \le \varepsilon /4$ with exponentially small exceptional asymptotic frequency at most $\rho$ with

$$\rho (\varepsilon ,\omega )= 2D(\varepsilon ,\omega ) 2\exp (-L 2^{-1} \varepsilon _b^2) = 4D(\varepsilon ,\omega ) \exp \left( -L \frac{450\varepsilon ^2}{\alpha ^2\sup _{t\in \mathbb {N}}\Vert h(\theta ^t\omega ,\cdot ,\cdot )\Vert ^2_{C^4}}\right) ,$$

where $$D(\varepsilon ,\omega ) = \max \{D_1(\varepsilon ,\omega ),D_2(\varepsilon ,\omega )\}\asymp \varepsilon ^{-1} \sup _{t\in \mathbb {N}}\Vert h(\theta ^t\omega ,\cdot ,\cdot )\Vert _{C^4}$$. $$\square $$

##### Proof of Theorem 2.4

Fix, as in the hypotheses of Theorem 2.4,

$$\varepsilon \ge \max \left\{ \alpha L^{-1/2},4\alpha \sup _{t\in \mathbb {N}}|h(\theta ^t \omega ,\cdot ,\cdot )|_{\textrm{Lip}} \varepsilon _s \left( \alpha L^{-1} \sup _{t\in \mathbb {N}} \Vert h(\theta ^t\omega ,\cdot ,\cdot )\Vert _{C^0} \right) \right\} ,$$

so that the (decoupling) term $\left| \zeta _d(t,\omega ,x,{{s}},x_{{s}})\right| \le \varepsilon /4$ by Lemma 2.12. Now fix $D(\varepsilon ,\omega ) = \max \{D_1(\varepsilon ,\omega ),D_2(\varepsilon ,\omega )\}$, so that (HFM) term $\left| \zeta _h(t,\omega ,x,\omega _{{s}},x_{{s}})\right| \le \varepsilon /2$ by Lemma 2.13 and (LFM) $\left| \zeta _{\ell }(t,\omega ,x,\omega _{{s}},x_{{s}})\right| \le \varepsilon /4$ with exponentially small exceptional asymptotic frequency at most $\rho$ with

$$\begin{aligned} \rho (\varepsilon ,\omega )= 4D(\varepsilon ,\omega ) \exp \left( -L \frac{450\varepsilon ^2}{\alpha ^2\sup _{t\in \mathbb {N}}\Vert h(\theta ^t\omega ,\cdot ,\cdot )\Vert ^2_{C^4}}\right) , \end{aligned}$$

by Lemma 2.14. It is also with this exponentially small exceptional asymptotic frequency $\rho$ that we conclude

$$\begin{aligned} \left| \xi _{m}(\theta ^t\omega ,x^t)\right|&\le \left| \zeta _d(t,\omega ,x,\omega _{{s}},x_{{s}})\right| +\left| \zeta _h(t,\omega ,x,\omega _{{s}},x_{{s}})\right| + \left| \zeta _{\ell }(t,\omega ,x,\omega _{{s}},x_{{s}})\right| \\&\le \varepsilon /4 + \varepsilon /2 + \varepsilon /4 = \varepsilon \end{aligned}$$

$$\square $$

##### Remark 2.15

In our estimates we control the fluctuation by shadowing in $|\zeta _d|\le \varepsilon /4$, decay of Fourier coefficients in $|\zeta _h|\le \varepsilon /2$, and rare visits to the bad set in $|\zeta _{\ell }|\le \varepsilon /4$. The choice to split $\varepsilon$ into these three portions has an impact on the outcome of constants such as $D(\varepsilon ,\omega )$, $c(\omega )$ and $\rho$. The choice of $\varepsilon _b$ in (13) impacts the exceptional asymptotic frequency $\rho$. Such choices are made only for convenience to illustrate the scaling relation among system size *L*, fluctuation size $\varepsilon$, and exceptional asymptotic frequency $\rho$.

### Reduction on a locally star-like network

As illustrated for the power-law network in the introduction, a hub looks locally like a star, in the sense that most of its neighbors are low degree nodes. In this section, we provide a definition to quantify this local feature.

#### Definition 2.16

*(Locally star-like network)* Let $G=(V,E)$ be an undirected graph on *N* nodes $V=\{1,\cdots ,N\}$ indexed so that the degree sequence is non-increasing $k_1\ge \cdots \ge k_N$.

Choose $\Delta \in \mathbb {N}$ as the hub scale and let $\mathcal {H}_{\Delta }:=\{i\in V: k_i\ge \Delta \}=\{1,\cdots ,M\}$ denote the collection of *M* hubs. Choose $\delta \in \mathbb {N}$ as the low degree scale and let $\mathcal {L}_{\delta }:=\{i\in V: k_i\le \delta \}=\{N-L+1,\cdots ,N\}$ denote the collection of *L* low degree nodes. We say that *G* is a $(\Delta ,\delta ,\nu )$-*locally star-like network* if most hub neighbors are low degree nodes in the sense that

$$\nu _i:= \frac{\# \mathcal {N}_i\cap \mathcal {L}_{\delta }}{\#\mathcal {N}_i} \ge \nu ,~~~~\forall i\in \mathcal {H}_{\Delta },$$

where $\mathcal {N}_i:= \{j\in V: A_{ij}=1\}$ denotes the neighbors of node *i*.

#### Remark 2.17

The star on *L* low degree nodes and one hub is locally star-like with $(\Delta ,\delta ,\nu )=(L,1,1)$. In a heterogeneous network, we will typically have $\nu$ near 1 and that the low degree scale $\delta$ is dominated by the hub degree scale $\Delta$ in the sense that $\delta /\Delta \rightarrow 0$ as $N\rightarrow +\infty$. There may be intermediate nodes that are neither hubs nor low degree nodes.

Now let *G* be a $(\Delta ,\delta ,\nu )$-locally star-like network on *N* nodes and consider *G*-network dynamics (7). The mean-field reduction seeks to approximate the mass action of the low-degree neighbors $\mathcal {N}_i\cap \mathcal {L}_{\delta }$ of a hub $z_i$, $i\in \mathcal {H}_{\Delta }$ as a space average against some probability measure *m* on $\mathbb {T}$

$$\alpha \frac{\nu _ik_i}{\Delta _0} \frac{1}{\nu _ik_i}\sum _{j=1}^N A_{ij}h(\omega ,z_i,x_j) = \alpha \frac{\nu _i k_i}{\Delta _0} \int _{\mathbb {T}} h(\omega ,z_i,y)\textrm{d}m(y)+\xi _{i,m}(\omega ,x),$$

where the fluctuation $\xi _{i,m}(\omega ,x)$ is given by

$$\alpha ^{-1}\xi _{i,m}(\omega ,x):= \frac{1}{\Delta _0} \sum _{j=1}^N A_{ij}h(\omega ,z_i,x_j) - \frac{\nu _i k_i}{\Delta _0} \int _{\mathbb {T}} h(\omega ,z_i,y)\textrm{d}m(y).$$

#### Theorem 2.18

(Reduction theorem on a locally star-like network) Suppose (R1–3) hold for the *G*-network random dynamical system (7) on a $(\Delta ,\delta ,\nu )$-locally star-like network *G* on *N* nodes. Let initial data $(\omega ,x)\in \Omega \times \mathbb {T}^{N}$ be such that the network trajectory $\{x^t=\Phi _{\alpha }(t,\omega ,x): t\in \mathbb {N}\}$ admits an *m*-typical shadowing orbit in the low degree coordinates; more precisely, there is $(\omega _{{s}},x_{{s}})\in \Omega \times \mathbb {T}^{N}$ satisfying

Shadowing $$\begin{aligned} \sup _{t\in \mathbb {N}}\max _{j\in \mathcal {L}_{\delta }} d_{\mathbb {T}}(x_j^t, \varphi _j(t,\omega _{{s}},x_{{s},j}))\le \varepsilon _s \left( \alpha \Delta _0^{-1} \delta \sup _{t\in \mathbb {N}} \Vert h(\theta ^t\omega ,\cdot ,\cdot )\Vert _{C^0}\right) ; \end{aligned}$$

Typicality $$\begin{aligned} \frac{1}{T}\sum _{t=0}^{T-1} \bigotimes _{j\in \mathcal {L}_{\delta }} \delta _{\varphi _j(t,\omega _{{s}},x_{{s},j})}\xrightarrow [T\rightarrow +\infty ]{\text {weak}^*} m^{\otimes L}, \end{aligned}$$

where $d_{\mathbb {T}}$ denotes the distance on the circle, the shadowing precision $\delta _s\mapsto \varepsilon _s (\delta _s )$ is a $\mathbb {R}_+$-valued function converging to 0 as $\delta _s$ tends to 0. Then, for any error tolerance

$$ \varepsilon \ge \max \left\{ \alpha \Delta ^{-1/2}, 4\alpha \sup _{t\in \mathbb {N}}|h(\theta ^t\omega ,\cdot ,\cdot )|_{\textrm{Lip}} \varepsilon _s \left( \alpha \Delta _0^{-1} \delta \sup _{t\in \mathbb {N}} \Vert h(\theta ^t\omega ,\cdot ,\cdot )\Vert _{C^0}\right) \right\} , $$

each hub $i\in \mathcal {H}_{\Delta }$ admits $(\varepsilon + \alpha (1-\nu )\sup _{t\in \mathbb {N}} \Vert h(\theta ^t\omega ,\cdot ,\cdot )\Vert _{C^0})$-reduction to $\varphi _{\alpha _i,m}$ in Eq. (12) with $\alpha _i=\alpha \frac{\nu _i\kappa _i}{\Delta _0}$ on initial data $(\omega ,x)\in \Omega \times \mathbb {T}^N$ with exceptional asymptotic frequency at most $\rho$ with

$$\begin{aligned} \rho (\varepsilon ,\omega )= &  4MD(\varepsilon ,\omega ) \exp (-\nu \Delta \varepsilon ^2 \alpha ^{-2} c(\omega )),~D(\varepsilon ,\omega )\asymp \varepsilon ^{-1}\sup _{t\in \mathbb {N}}\Vert h(\theta ^t\omega ,\cdot ,\cdot )\Vert _{C^4}, \\ c(\omega )= &  \frac{450}{\sup _{t\in \mathbb {N}}\Vert h(\theta ^t\omega ,\cdot ,\cdot )\Vert ^2_{C^4}}, \end{aligned}$$

where *M* denotes the number of hubs in *G* and the constants $D(\varepsilon ,\omega )$ and $c(\omega )$ are the same as in Theorem 2.4 independent of *N* and *x*.

We will prove this theorem in a similar way as we did the reduction on a star Theorem 2.4. The main modification concerns the bad sets for multiple hubs and the control of non-low-degree neighbors of the hubs.

#### Proof of reduction theorem on a locally star-like network

##### Proof of Theorem 2.18

We wiill split the fluctuation $\xi _{i,m}(\theta ^t\omega ,x^t)$ at hub $i\in \mathcal {H}_{\Delta }$

$$\begin{aligned} \xi _{i,m}(\theta ^t\omega ,x^t)&= \frac{\alpha }{\Delta _0} \sum _{j\in \mathcal {N}_i} h(\theta ^t\omega ,z_i^t,x_j^t) - \alpha \frac{\nu _i k_i}{\Delta _0}\int _{\mathbb {T}} h(\theta ^t\omega ,z_i^t,y)\textrm{d}m(y) \end{aligned}$$

into four components. By (Shadowing), the first term

decoupling $$\begin{aligned} \zeta _{i,d}(t,\omega ,x,\omega _{{s}},x_{{s}})&:= \frac{\alpha }{\Delta _0} \sum _{j\in \mathcal {N}_i\cap \mathcal {L}_{\delta }} \left[ h(\theta ^t\omega ,z_i^t,x_j^t) - h(\theta ^t\omega ,z_i^t,x_{{s},j}^t) \right] \end{aligned}$$

decouples the low-degree node orbits $x_j^t=\Phi _{\alpha }(t,\omega ,x)_j, j\in \mathcal {N}_i\cap \mathcal {L}_{\delta }$ by comparing with the isolated shadowing orbits $x_{{s},j}^t =\varphi _j(t,\omega _{{s}},x_{{s},j})$, which will be controlled by the shadowing precision and Lipschitz continuity of $h(\theta ^t\omega ,\cdot ,\cdot )$, see Lemma 2.19. The second term gathers contribution from the non-low-degree neighbors

non ldn $$\begin{aligned} \zeta _{i,c}(t,\omega ,x,\omega _{{s}},x_{{s}})&:= \frac{\alpha }{\Delta _0} \sum _{j\in \mathcal {N}_i\setminus \mathcal {L}_{\delta }} h(\theta ^t\omega ,z_i^t,x_j^t), \end{aligned}$$

which will be controlled by the star-like index $\frac{\# \mathcal {N}_i\setminus \mathcal {L}_{\delta }}{\Delta _0}\le 1-\nu \approx 0$, see Lemma 2.20. This is a new term that was not present in the proof of Theorem 2.4. Now we have

$$\xi _{i,m}(\theta ^t\omega ,x^t) \le \zeta _{i,d} + \zeta _{i,c} + \alpha \frac{\nu _1k_1}{\Delta _0}\left[ \frac{1}{\nu _ik_i}\sum _{j\in \mathcal {N}_i\cap \mathcal {L}_{\delta }}h(\theta ^t\omega ,z_i^t,x_{{s},j}^t) -\int _{\mathbb {T}}h(\theta ^t\omega ,z_i^t,y)\textrm{d}m(y)\right] .$$

By (Typicality) the shadowing orbit $\left( x_{{s},j}^t\right) _{j\in \mathcal {L}_{\delta }}$ distributes in time as $m^{\otimes L}$ and thus visits any bad set with controlled asymptotic frequency. To find the appropriate bad sets, we take Fourier series expansion for $h(\omega ,x_1,x_2)$ and note that the Fourier modes corresponding to $n_2=0$ drop out, similar to the proof of Theorem 2.4.

Again we gather the remaining high Fourier modes $\phi _{n_2}$ with $|n_2|>D$ above some cutoff level *D*

HFM $$\begin{aligned} \zeta _{i,h}(t,\omega ,x,\omega _{{s}},x_{{s}})&:= \alpha \frac{\nu _i k_i}{\Delta _0}\sum _{|n_2|>D} \left[ a_{(0,n_2)}^{\theta ^t\omega } + \sum _{n_1\ne 0} a_{(n_1,n_2)}^{\theta ^t\omega } \phi _{n_1}(z_i^t) \right] \\&\quad \left[ \frac{1}{\nu _i k_i} \sum _{j\in \mathcal {N}_i\cap \mathcal {L}_{\delta }}\phi _{n_2}(x_{{s},j}^t) - \int _{\mathbb {T}} \phi _{n_2}(y) \textrm{d}m(y)\right] , \end{aligned}$$

which will be controlled by decay of Fourier coefficients, see Lemma 2.21. Lastly, the low Fourier modes $\phi _{n_2}$ with $1\le |n_2|\le D$ make up the last term

LFM $$\begin{aligned} \zeta _{i,\ell }(t,\omega ,x,\omega _{{s}},x_{{s}})&:= \alpha \frac{\nu _i k_i}{\Delta _0}\sum _{1\le |n_2|\le D} \left[ a_{(0,n_2)}^{\theta ^t\omega } + \sum _{n_1\ne 0} a_{(n_1,n_2)}^{\theta ^t\omega } \phi _{n_1}(z_i^t) \right] \\&\quad \left[ \frac{1}{\nu _i k_i} \sum _{j\in \mathcal {N}_i\cap \mathcal {L}_{\delta }}\phi _{n_2}(x_{{s},j}^t) - \int _{\mathbb {T}} \phi _{n_2}(y) \textrm{d}m(y)\right] , \end{aligned}$$

which will be controlled by rare visits of the shadowing orbit $\left( x_{{s},j}^t\right) _{j\in \mathcal {L}_{\delta }}$ to the bad sets, to be defined for each hub $i\in \mathcal {H}_{\Delta }$, see Lemma 2.22. In the next four lemmas, we estimate each of $\zeta _{i,d},\zeta _{i,c},\zeta _{i,h},\zeta _{i,\ell }$. $\square$

##### Lemma 2.19

(Shadowing estimates for low degree nodes). Under the hypotheses of Theorem 2.18, we control the $\mathrm {(decoupling)}$ term

$$ |\zeta _{i,d}(t,\omega ,x,\omega _{{s}},x_{{s}})|\le \varepsilon /4,~~~~\forall i\in \mathcal {H}_{\Delta },~~\forall t\in \mathbb {N}. $$

##### Proof

The estimate follows by assumption of Theorem 2.18 similar to the proof of Lemma 2.12. $\square$

##### Lemma 2.20

(Non low degree nodes estimates). Under the hypotheses of Theorem 2.18, we control the contribution $\mathrm {(non ~ldn)}$ from non low degree neighbors

$$ |\zeta _{i,c}(t,\omega ,x,\omega _{{s}},x_{{s}})|\le \alpha (1-\nu )\sup _{t\in \mathbb {N}} \Vert h(\theta ^t\omega ,\cdot ,\cdot )\Vert _{C^0},~~~~\forall i\in \mathcal {H}_{\Delta },~~\forall t\in \mathbb {N}. $$

##### Proof

follows directly from the $(\Delta ,\delta ,\nu )$-locally star-like properties of *G*. $\square$

##### Lemma 2.21

(High Fourier modes controlled by decay of Fourier coefficients). Under the hypotheses of Theorem 2.18, by choosing

$$ D(\varepsilon ,\omega ) \asymp \varepsilon ^{-1}\sup _{t\in \mathbb {N}}\Vert h(\theta ^t\omega ,\cdot ,\cdot )\Vert _{C^4} $$

as in Lemma 2.13, we estimate $\mathrm {(HFM)}$

$$ |\zeta _{i,h}(t,\omega ,x,\omega _{{s}},x_{{s}})|\le \varepsilon /2,~~~~\forall i\in \mathcal {H}_{\Delta },~~\forall t\in \mathbb {N}. $$

##### Proof

The estimates are similar to Lemma 2.13 with $\alpha$ replaced by $\alpha _i = \alpha \frac{\nu _i\kappa _i}{\Delta _0}$. $\square$

##### Lemma 2.22

(Low Fourier modes controlled by rare visits to the bad sets). Under the hypotheses of Theorem 2.18, by choosing

$$D(\varepsilon ,\omega ) \asymp \varepsilon ^{-1} \sup _{t\in \mathbb {N}}\Vert h(\theta ^t\omega ,\cdot ,\cdot )\Vert _{C^4},$$

we control $$\mathrm {(LFM)}$$

$$ |\zeta _{i,\ell }(t,\omega ,x,\omega _{{s}},x_{{s}})|\le \varepsilon /4,~~~~\forall i\in \mathcal {H}_{\Delta } $$

with exponentially small exceptional asymptotic frequency at most $\rho$ with

$$\rho (\varepsilon ,\omega )= 4MD(\varepsilon ,\omega ) \exp \left( -\nu \Delta \frac{450\varepsilon ^2}{\alpha ^2\sup _{t\in \mathbb {N}}\Vert h(\theta ^t\omega ,\cdot ,\cdot )\Vert ^2_{C^4}} \right) .$$

##### Proof

We estimate $|\zeta _{i,\ell }(t,\omega ,x,\omega _{{s}},x_{{s}})|$ as a problem of frequency of visits to the bad sets

$$B_{i,n}= B_i(\varepsilon _b, \phi _n):=\left\{ x\in \mathbb {T}^N: \left| \frac{1}{\nu _i k_i}\sum _{j\in \mathcal {N}_i\cap \mathcal {L}_{\delta }} \phi _n(x_j) - \int _{\mathbb {T}} \phi _n(y)\textrm{d}m(y)\right| >\varepsilon _b\right\} ,$$

for $i\in \mathcal {H}_{\Delta }$ and $n=\pm 1,\cdots ,\pm D$, totaling 2*MD* bad sets. By a similar argument to that for Size of Bad Sets Proposition 2.9, we use Hoeffding inequality to obtain estimates for each of them

$$m^{\otimes N} (B_{i,n}) \le 2\exp (-\nu _i k_i2^{-1} \Vert \phi _{n}\Vert _{C^0}^{-2} \varepsilon _b^2) \le 2\exp (-\nu \Delta 2^{-1}\varepsilon _b^2).$$

An argument similar to Lemma 2.11 implies that the shadowing orbit $x_{{s}}^t = (\varphi _{N-L+1}(t,\omega _{{s}}, x_{{s},N-L+1}),\cdots ,\varphi _{N}(t,\omega _{{s}}, x_{{s},N}))$ visits each $B_{i,n}$ with exponentially small frequency at most $2\exp (-\nu \Delta 2^{-1} \varepsilon _b^2)$. Whenever all such bad sets are avoided, we estimate

$$\begin{aligned} |\zeta _{i,\ell }(t,\omega ,x,\omega _{{s}},x_{{s}})|&\le \alpha \frac{\nu _i k_i}{\Delta _0}\sum _{1\le |n_2|\le D} \left[ |a_{(0,n_2)}^{\theta ^t\omega }| + \sum _{n_1\ne 0} |a_{(n_1,n_2)}^{\theta ^t\omega }| \right] \left| \frac{1}{\nu _i k_i} \sum _{j\in \mathcal {N}_i\cap \mathcal {L}_{\delta }}\phi _{n_2}(x_{{s},j}^t) \right. \\&\quad \left. - \int _{\mathbb {T}} \phi _{n_2}(y) \textrm{d}m(y)\right| \\&\le \alpha \sum _{1\le |n_2|\le D} \left[ \frac{\sup _{t\in \mathbb {N}}\Vert h(\theta ^t\omega ,\cdot ,\cdot )\Vert _{C^4}}{(2\pi )^4 n_2^4} + \sum _{n_1\ne 0} \frac{\sup _{t\in \mathbb {N}}\Vert h(\theta ^t\omega ,\cdot ,\cdot )\Vert _{C^4}}{(2\pi )^4 n_1^2n_2^2} \right] \varepsilon _b\\&\le \frac{\alpha \sup _{t\in \mathbb {N}}\Vert h(\theta ^t\omega ,\cdot ,\cdot )\Vert _{C^4}}{(2\pi )^4 } \left[ \frac{2\pi ^4}{90} + \frac{2\pi ^2}{6}\frac{2\pi ^2}{6} \right] \varepsilon _b \\&= \frac{\alpha \sup _{t\in \mathbb {N}}\Vert h(\theta ^t\omega ,\cdot ,\cdot )\Vert _{C^4}}{120 } \varepsilon _b. \end{aligned}$$

By choosing

$$\begin{aligned}&\varepsilon _b=\frac{30}{\alpha \sup _{t\in \mathbb {N}}\Vert h(\theta ^t\omega ,\cdot ,\cdot )\Vert _{C^4}}\varepsilon ,\\&D(\varepsilon ,\omega )= \max \{D_1(\varepsilon ,\omega ),D_2(\varepsilon ,\omega )\}\asymp \varepsilon ^{-1} \sup _{t\in \mathbb {N}}\Vert h(\theta ^t\omega ,\cdot ,\cdot )\Vert _{C^4},\end{aligned}$$

we obtain $|\zeta _{i,\ell }(t,\omega ,x,\omega _{{s}},x_{{s}})| \le \varepsilon /4$ with exponentially small exceptional frequency at most $\rho$ with

$$\begin{aligned} \rho (\varepsilon ,\omega )=4MD(\varepsilon ,\omega ) \exp (-\nu \Delta 2^{-1} \varepsilon _b^2) = 4MD(\varepsilon ,\omega ) \exp \left( -\nu \Delta \frac{450\varepsilon ^2}{\alpha ^2\sup _{t\in \mathbb {N}}\Vert h(\theta ^t\omega ,\cdot ,\cdot )\Vert ^2_{C^4}}\right) . \end{aligned}$$

##### Proof of Theorem 2.18

Fix, as in the hypotheses of Theorem 2.18,

$$\varepsilon \ge \max \left\{ \alpha \Delta ^{-1/2}, 4\alpha \varepsilon _s \left( \alpha \Delta _0^{-1} \delta \sup _{t\in \mathbb {N}} \Vert h(\theta ^t\omega ,\cdot ,\cdot )\Vert _{C^0}\right) \sup _{t\in \mathbb {N}}|h(\theta ^t\omega ,\cdot ,\cdot )|_{\textrm{Lip}}\right\} , $$

so that the (decoupling) term $\left| \zeta _{i,d}(t,\omega ,x,{{s}},x_{{s}})\right| \le \varepsilon /4$ for all $i\in \mathcal {H}_{\Delta }$ and $t\in \mathbb {N}$ by Lemma 2.19. The locally star-like assumption of Theorem 2.18 yields (non ldn) $|\zeta _{i,c}(t,\omega ,x,\omega _{{s}},x_{{s}})|\le \alpha (1-\nu )\sup _{t\in \mathbb {N}} \Vert h(\theta ^t\omega ,\cdot ,\cdot )\Vert _{C^0}$ for all $i\in \mathcal {H}_{\Delta }$ and $t\in \mathbb {N}$ by Lemma 2.20. Now fix $D(\varepsilon ,\omega ) = \max \{D_1(\varepsilon ,\omega ),D_2(\varepsilon ,\omega )\}$, so that (HFM) term $\left| \zeta _{i,h}(t,\omega ,x,\omega _{{s}},x_{{s}})\right| \le \varepsilon /2$ for all $i\in \mathcal {H}_{\Delta }$ and $t\in \mathbb {N}$ by Lemma 2.21 and (LFM) $\left| \zeta _{i,\ell }(t,\omega ,x,\omega _{{s}},x_{{s}})\right| \le \varepsilon /4$ for all $i\in \mathcal {H}_{\Delta }$ and with exponentially small exceptional asymptotic frequency at most $\rho$ with

$$\rho (\varepsilon ,\omega )= 4MD(\varepsilon ,\omega ) \exp \left( -\nu \Delta \frac{450\varepsilon ^2}{\alpha ^2\sup _{t\in \mathbb {N}}\Vert h(\theta ^t\omega ,\cdot ,\cdot )\Vert ^2_{C^4}} \right) .$$

by Lemma 2.22. It is also with this exponentially small exceptional asymptotic frequency $\rho$ that we conclude

$$\begin{aligned} \max _{i\in \mathcal {H}_{\Delta }}\left| \xi _{i,m}(\theta ^t\omega ,x^t)\right|&\le \sum _{k=d,c,h,\ell } \max _{i\in \mathcal {H}_{\Delta }}\left| \zeta _{i,k}(t,\omega ,x,\omega _{{s}},x_{{s}})\right| \\&\le \varepsilon + \alpha (1-\nu )\sup _{t\in \mathbb {N}} \Vert h(\theta ^t\omega ,\cdot ,\cdot )\Vert _{C^0} \end{aligned}$$

$$\square $$

## Examples

In this section we provide a general class of examples of node dynamics, coupling functions and network structures satsifying the dimensional reduction Theorems 2.4 and 2.18.

### Node dynamics satisfying shadowing and typicality

Consider the node dynamics given by the iid random iteration $\varphi$ of a measurable family of uniform contractions on a compact metric space (*M*, *d*), endowed with its Borel $\sigma$-algebra $\mathcal {B}(M)$. More precisely, over a probability preserving transformation $(\Omega ,\mathcal {F},\mathbb {P},\theta )$, let

14 $$\begin{aligned} \varphi :\mathbb {N}\times \Omega \times M\rightarrow M \end{aligned}$$

satisfy:

1. measurability with respect to $2^{\mathbb {N}}\otimes \mathcal {F}\otimes \mathcal {B}(M)$ and $\mathcal {B}(M)$;
2. cocycle property over $\theta$: $\varphi (0,\omega ,\cdot )=\textrm{id}_M$ for all $\omega \in \Omega$ and $\varphi (t,\theta ^s\omega ,\cdot )\circ \varphi (s,\omega ,\cdot )= \varphi (t+s,\omega ,\cdot )$ for all $s,t\in \mathbb {N}$ and $\omega \in \Omega$;
3. the maps $\varphi (1,\theta ^t\omega ,\cdot )$ have independent and identical distribution;
4. there is a uniform contraction rate $\lambda \in (0,1)$ for which
  $$d(\varphi (1,\omega ,x),\varphi (1,\omega ,y))\le \lambda d(x,y),~~~~\forall \omega \in \Omega , x,y\in M.$$

Note that $\varphi$ is a continuous RDS over $\sigma$ in the sense of Arnold [Arn98].

#### Unique stationary measure

The iid random iteration $\varphi$ in (14) induces, see [Arn98, Theorem 2.1.4], the Markov chain

15 $$\begin{aligned} Z_{n+1}(\omega ):= \varphi (1,\theta ^{n}\omega ,Z_{n}(\omega )),~~~~n=0,1,\cdots , \end{aligned}$$

where $Z_0$ is independent of the random contractions $\varphi (1,\theta ^t\omega ,\cdot )$ with transition probability

$$P(x,B)=\mathbb {P}(\omega \in \Omega : \varphi (1,\omega ,x)\in B).$$

Note that the transition probability *P*(*x*, *B*) acts on measures $\mu \in \mathcal {M}_1(M,\mathcal {B}(M))$ by

$$\mu \mapsto \mu P,~~~~\mu P(B):=\int _M P(x,B)\textrm{d}\mu (x),~~\forall B\in \mathcal {B}(M),$$

and acts on bounded measurable functions $g\in b(M;\mathbb {R})$ by

$$g\mapsto Pg,~~~~Pg(x):=\int _M g(y) P(x,\textrm{d}y),~~~~\forall x\in M.$$

These two actions are in dual relation with each other

$$\int _M g \textrm{d}\mu P = \int _M Pg \textrm{d}\mu ,~~~~\forall g\in b(M;\mathbb {R}),\mu \in \mathcal {M}_1(M,\mathcal {B}(M)).$$

A *stationary measure* *m* is defined by the property $mP=m$, or

$$\int _M P(x,B)\textrm{d}m(x)=m(B),~~~~\forall B\in \mathcal {B}(M).$$

Define the *coding map* by pre-composition

16 $$\begin{aligned} \pi :\Omega \rightarrow M,~~~~\pi (\omega ):=\lim _{n\rightarrow +\infty } \varphi (1,\omega ,\cdot )\circ \varphi (1,\theta \omega ,\cdot )\circ \cdots \circ \varphi (1,\theta ^{n-1}\omega ,p),~~p\in M, \end{aligned}$$

where the limit exists and is independent of the choice of initial point $p\in M$. For well-definedness of the coding map $\pi$, we have used the facts that $\varphi (1,\omega ,\cdot )$ is a family of contractions with uniform rate $\lambda \in (0,1)$ and that *M* is a compact metric space; note that the family $\{\varphi (1,\omega ,\cdot ):\omega \in \Omega \}$ of contractions can be finite, countable or uncountable. By Letac principle [Let86], the coding map implies the existence and uniqueness of stationary measure *m* for the Markov chain (15).

##### Proposition 3.1

The Markov chain (15) admits a unique stationary measure $\pi _*\mathbb {P}$ on *M*.

#### Typical random orbits

Now we will establish a very strong sense of typicality for the random orbits of an iid random iteration of uniform contractions on a compact metric space.

##### Proposition 3.2

Let $\varphi$ be an iid random iteration of continuous maps on compact metric state space *M* and let $\{Z_n:n\in \mathbb {N}\}$ be the induced Markov chain. Then, with probability one, any accumulation point of the sequence $\frac{1}{N}\sum _{n=0}^{N-1} \delta _{Z_n}$ in $\mathcal {M}^1(M)$ endowed with the weak-star topology is a stationary measure.

##### Proof

In light of Breiman’s Ergodic Theorem [Bre60], we only need to verify that $x\mapsto P(x,\cdot )$ is continuous in the weak-star topology on $\mathcal {M}^1(M)$. Since *M* is a compact metric space, in particular, sequential, it suffices to check that $x_k\rightarrow x$ in *M* implies $P(x_k,\cdot )\rightarrow P(x,\cdot )$ in $\mathcal {M}^1(M)$. For this, fix any $g\in C(M)\subseteq b(M,\mathcal {B})$. It follows from RDS theory [Arn98, Theorem 2.1.4] that the transition probability *P* is Feller, and in the particular case of compact state space *M*, we conclude *Pg* is continuous. Now we have

$$\begin{aligned}&P(x_k,g) = \int _M g(y) P(x_k,\textrm{d}y) = (Pg)(x_k)\xrightarrow {k\rightarrow +\infty } (Pg)(x)=P(x,g)~~~~\\&\text {because }Pg\in C(M), \end{aligned}$$

as required. $$\square $$

##### Theorem 3.3

(Uniform typicality of random orbits) Consider the iid random iteration $\varphi$ of uniform contractions given in (14) satisfying (C1-4). There is a uniform set $\Omega _*\subseteq \Omega$ of full $\mathbb {P}$-measure such that for every initial condition $x\in M$ and every noise $\omega \in \Omega _*$, the asymptotic behavior of the random orbit $\varphi (t,\omega ,x)$ is described by the stationary measure

$$\frac{1}{T} \sum _{t=0}^{T-1} \delta _{\varphi (t,\omega ,x)}\xrightarrow [T\rightarrow +\infty ]{\text {weak}^*} \pi _{*}\mathbb {P},$$

where $\pi :\Omega \rightarrow M$ denotes the coding map given in (16).

##### Remark 3.4

By assuming additionally that $\Omega$ is a compact metric space, [MM20] obtains a similar type of uniform typicality for a class of iid random iteration of continuous maps strongly synchronizing on average. Also, by arguments similar to the one presented below, one can establish the uniform typicality for a class of so-called *J*-monotone maps on a compact connected subset of $\mathbb {R}^k$, see [MS21, Theorem 2].

##### Proof

Consider the Markov chain $\{Z_n\}$ in (15) for some deterministic initial condition $x_0\in M$. By uniqueness of stationary distribution $\pi _{*}\mathbb {P}$ from Proposition 3.1, we obtain from Proposition 3.2$\Omega _{x_0}\subseteq \Omega$ with $\mathbb {P}(\Omega _{x_0})=1$ for which

$$\frac{1}{T} \sum _{t=0}^{T-1} \delta _{\varphi (t,\omega ,x_0)}= \frac{1}{T} \sum _{t=0}^{T-1} \delta _{Z_t(\omega )}\xrightarrow [T\rightarrow +\infty ]{\text {weak}^*} \pi _{*}\mathbb {P},~~~~\forall \omega \in \Omega _{x_0}.$$

Now fix any $\omega \in \Omega _*:=\Omega _{x_0}$, $x\in M$, $\varepsilon >0$, and $h\in C^0(M)$. We show that there is some $T_0$ for which

$$T\ge T_0~~\Rightarrow ~~\left| \left( \frac{1}{T} \sum _{t=0}^{T-1} \delta _{\varphi (t,\omega ,x)}\right) (h) - \pi _{*}\mathbb {P}(h)\right| \le \varepsilon .$$

Indeed,

$$\begin{aligned}&\left| \left( \frac{1}{T} \sum _{t=0}^{T-1} \delta _{\varphi (t,\omega ,x)}\right) (h) - \pi _{*}\mathbb {P}(h)\right| \\&\quad \le \left| \left( \frac{1}{T} \sum _{t=0}^{T-1} \delta _{\varphi (t,\omega ,x)}\right) (h) - \left( \frac{1}{T} \sum _{t=0}^{T-1} \delta _{\varphi (t,\omega ,x_0)}\right) (h) \right| + \left| \left( \frac{1}{T} \sum _{t=0}^{T-1} \delta _{\varphi (t,\omega ,x_0)}\right) (h) - \pi _{*}\mathbb {P}(h)\right| \\&\quad \le \frac{1}{T} \sum _{t=0}^{T-1} |h(\varphi (t,\omega ,x)) - h(\varphi (t,\omega ,x_0))| + \left| \left( \frac{1}{T} \sum _{t=0}^{T-1} \delta _{\varphi (t,\omega ,x_0)}\right) (h) - \pi _{*}\mathbb {P}(h)\right| \end{aligned}$$

By continuity and hence uniform continuity of *h* on *M*, there is $\delta (\varepsilon )>0$ such that $d_M(x,y)\le \delta (\varepsilon )$ implies $|h(x)-h(y)|\le \varepsilon$. Also, since $\textrm{diam} f_{\omega }^t(M) \le \lambda ^t \textrm{diam} M\rightarrow 0$, it follows that there is some $T_1(\delta )$ such that $t\ge T_1(\delta )$ implies $\sup _{x,y\in M}d_M(\varphi (t,\omega ,x),\varphi (t,\omega ,y))\le \delta$. For $T_2(\varepsilon )=T_1(\delta (\varepsilon /3))$, we have

$$d_M(\varphi (t,\omega ,x) , \varphi (t,\omega ,x_0) )\le \delta (\varepsilon /3),~~~~\forall t\ge T_2(\varepsilon )$$

and hence

$$|h(\varphi (t,\omega ,x)) - h(\varphi (t,\omega ,x_0)) |\le \varepsilon /3.~~~~\forall t\ge T_2(\varepsilon ).$$

Choose $T_3(\varepsilon )$ for which

$$\frac{1}{T}T_2(\varepsilon )2\Vert h\Vert _{C^0}\le \varepsilon /3,~~~~\forall T\ge T_3(\varepsilon ).$$

Combining the above two estimates, we obtain

$$\begin{aligned}&\frac{1}{T} \sum _{t=0}^{T-1} |h(\varphi (t,\omega ,x)) - h(\varphi (t,\omega ,x_0))|\\ &\quad \le \frac{1}{T}\sum _{t=0}^{T_2(\varepsilon )-1} |h(\varphi (t,\omega ,x)) - h(\varphi (t,\omega ,x_0))| + \frac{1}{T}\sum _{t=T_2(\varepsilon )}^{T-1} |h(\varphi (t,\omega ,x)) - h(\varphi (t,\omega ,x_0))| \\&\quad \le \frac{T_2(\varepsilon ) 2 \Vert h\Vert _{C^0} + (T-T_2(\varepsilon ))\varepsilon /3}{T} \le 2\varepsilon /3,~~~~\forall T\ge \max \{T_2(\varepsilon ),T_3(\varepsilon )\}. \end{aligned}$$

By weak-star convergence $\frac{1}{T} \sum _{t=0}^{T-1} \delta _{\varphi (t,\omega ,x_0)}\rightarrow \pi _{*}\mathbb {P}$, there is some $T_4(\varepsilon )$ for which

$$\left| \left( \frac{1}{T} \sum _{t=0}^{T-1} \delta _{\varphi (t,\omega ,x_0)}\right) (h) - \pi _{*}\mathbb {P}(h)\right| \le \varepsilon /3,~~~~\forall T\ge T_4(\varepsilon ).$$

We thus conclude that for $T\ge T_0:=\max \{T_2(\varepsilon ),T_3(\varepsilon ),T_4(\varepsilon )\}$, we have

$$\left| \left( \frac{1}{T} \sum _{t=0}^{T-1} \delta _{\varphi (t,\omega ,x)}\right) (h) - \pi _{*}\mathbb {P}(h)\right| \le \varepsilon ,$$

as required. $$\square $$

#### Shadowing and Typicality on the star

##### Corollary 3.5

Consider star network dynamics (9-10) satisfying (R1–3), where the node dynamics are given by iid random iteration of contractions $\varphi _i$ in (14) with uniform contraction rate $\lambda \in (0,1)$ and unique stationary measure $m_0:=\pi _*\mathbb {P}$ as in Theorem 3.3. Then, for any error tolerance

$$\varepsilon \ge \left\{ \alpha L^{-1/2},4\alpha ^2 L^{-1} (1-\lambda )^{-1}\sup _{t\in \mathbb {N}} \Vert h(\theta ^t\omega ,\cdot ,\cdot )\Vert _{C^0} \sup _{t\in \mathbb {N}} |h(\theta ^t\omega ,\cdot ,\cdot )|_{\textrm{Lip}}\right\} ,$$

the hub behavior admits $\varepsilon$-reduction to $\varphi _{\alpha ,m_0}$ defined in (12), for almost every noise realization $\omega$ and any initial condition $x\in \mathbb {T}^N$ with exponentially small exceptional asymptotic frequency at most $\rho$ with

$$\rho (\varepsilon ,\omega )= D(\varepsilon ,\omega )\exp (-L\varepsilon ^2 \alpha ^{-2}c(\omega )),$$

where $D(\varepsilon ,\omega )$ and $c(\omega )$ are the same constants independent of *N* and *x* as in Theorem 2.4.

##### Proof

By (R1) and (C4), the $\varphi _i$ are independent random uniform contractions on $\mathbb {T}$ with rate $\lambda$. It follows that the uncoupled system $\Phi _{\alpha =0}$ is a random uniform contraction satisfying (C4) on $\mathbb {T}^{N}$, equipped with the distance $d_{\mathbb {T}^{N}}(x,y)= \max _{i=1,\cdots ,N} d_{\mathbb {T}}(x_i,y_i)$, with the same rate $\lambda$. By Theorem 3.3 applied to $\Phi _{\alpha =0}$ on $(\mathbb {T}^{N},d_{\mathbb {T}^{N}})$, the random orbit $\Phi _{\alpha =0}(t,\omega ,x)$ starting from any $x\in \mathbb {T}^N$ and $\omega \in \Omega _*$ asymptotically distributes as the unique stationary measure $m_0^{\otimes N}$.

To verify the (Typicality) assumption in Theorem 2.4, simply take $x_s=x\in \mathbb {T}^N$ and $\omega _s=\omega \in \Omega _*$. For the (Shadowing) assumption, we compute

$$\begin{aligned} d_{\mathbb {T}}(x_j^t, \varphi _j(t,\omega ,x_j))&\le d_{\mathbb {T}}\left( \varphi _j(1,\theta ^{t-1}\omega ,x_j^{t-1}) + \frac{\alpha }{L} h(\theta ^{t-1}\omega ,x_j^{t-1},z^{t-1}), \right. \\&\quad \left. \varphi _j(1,\theta ^{t-1}\omega )\circ \varphi _j(t-1,\omega ,x_j)\right) \\&\le \frac{\alpha }{L} \sup _{t\in \mathbb {N}} \Vert h(\theta ^t\omega ,\cdot ,\cdot )\Vert _{C^0}+ \lambda \cdot d_{\mathbb {T}}(x_j^{t-1}, \varphi _j(t-1,\omega ,x_j))\\&\le \alpha L^{-1} \sup _{t\in \mathbb {N}} \Vert h(\theta ^t\omega ,\cdot ,\cdot )\Vert _{C^0}(1+\lambda + \cdot + \lambda ^{t-1}) \\&\le \alpha L^{-1} \sup _{t\in \mathbb {N}} \Vert h(\theta ^t\omega ,\cdot ,\cdot )\Vert _{C^0} \frac{1}{1-\lambda },~~~~\forall t\in \mathbb {N}. \end{aligned}$$

We have thus verified for almost every noise realization $\omega$ and for each initial condition $x\in \mathbb {T}^N$ (Shadowing) with shadowing precision $\varepsilon _s = \frac{1}{1-\lambda } \alpha L^{-1} \sup _{t\in \mathbb {N}} \Vert h(\theta ^t\omega ,\cdot ,\cdot )\Vert _{C^0}$.

The corollary follows from Theorem 2.4. $\square$

### A locally star-like random power-law graph model

We consider the expected degree sequence $\varvec{\textrm{w}}$ defined in (6). Then the actual and expected degrees are close:

#### Lemma 3.6

(Degree Concentration in *n*; [CL06] Lemma 5.7). For a graph *G* in $G(\varvec{\textrm{w}})$, with probability $1-n^{-1/5}$ all nodes *i* simultaneously satisfy

$$|k_i- w_i| \le 2 (\sqrt{w_i \log n} + \log n).$$

The Chung-Lu random power-law graph model is defined by (6) without any canonical scale choices for hub and low degree scales $\Delta ,\delta$. Therefore, we will first make more concrete choices of the parameters *m*, *w*, and then introduce the locally star-like parameters $(\Delta ,\delta ,\nu )$.

#### Theorem 3.7

(A locally star-like random power-law graph) Fix power-law exponent $\beta >2$. Consider a graph *G* in the Chung-Lu random power-law graph model defined by the expected degree sequence $\varvec{\textrm{w}}$ in (6) with

$$\begin{aligned} m&= c_{\textrm{hub}}\cdot n^{\frac{1}{\beta -1}},~~~~\text {for some }c_{\textrm{hub}}\asymp 1,\\ w&= o\left( n^{\frac{\lambda _{\textrm{ldn}}}{\beta -1}}\right) ,~~~~\text {for some }\lambda _{\textrm{ldn}}\in (0,1), \end{aligned}$$

and hub and low degree scales

$$\Delta \sim \lambda _{\textrm{hub}} m, ~~\text {for some } \lambda _{\textrm{hub}}\in (0,1);~~~~\delta \sim c_{\textrm{ldn}} m^{\lambda _{\textrm{ldn}}},~~\text {for some }c_{\textrm{ldn}}\asymp 1.$$

Then, with probability $1-O(n^{-1/5})$, we have

$$M\asymp w^{\beta -1},~~~~L\sim n,~~~~\nu =1-O\left( n^{-\lambda _{\textrm{ldn}}\frac{\beta -2}{\beta -1}} w^{\beta -2}\right) ,$$

where

(i) the first *M* nodes $1,\cdots ,M$ are hubs of degree at least $\Delta$,
(ii) the last *L* nodes $n-L+1,\cdots ,n$ are low degree nodes of degree at most $\delta$,
(iii) *G* is $(\Delta ,\delta ,\nu )$-locally star-like.

#### Definition 3.8

*(Locally star-like random power-law graph model)* We will call the model considered in Theorem 3.7 the *locally star-like random power-law graph model* and denote it by $\textrm{LSL}(\beta ,n)$.

#### Proof of Theorem 3.7

Our strategy is to count the number of nodes that are expected to be hubs in Step **I** and low degree nodes in Step **II**, prove the expected picture satisfies the locally star-like property in Step **III**, and finally bring the estimates to the actual degrees by concentration.

**I.** First we compute the number *M* of expected hubs by solving the equation $w_{M}=\lambda _{\textrm{hub}}\cdot m$, which reads

$$\begin{aligned} \frac{\beta -2}{\beta -1} w n ^{\frac{1}{\beta -1}} \cdot \left( n\left( \frac{w(\beta -2)}{m(\beta -1)}\right) ^{\beta -1} +M -1\right) ^{-\frac{1}{\beta -1}}&= \lambda _{\textrm{hub}}\cdot m. \end{aligned}$$

By multiplying $\left( \frac{\beta -2}{\beta -1} w n ^{\frac{1}{\beta -1}} \right) ^{-1}$ and taking the $-(\beta -1)$-power on both sides, we obtain

$$\begin{aligned} M&=1 + n \left( \frac{w(\beta -2)}{m(\beta -1)}\right) ^{\beta -1} (\lambda _{\textrm{hub}}^{-(\beta -1)} - 1). \end{aligned}$$

By the choices of *w* and *m*, we obtain

$$\begin{aligned}&M\sim n w^{\beta -1} (c_{\textrm{hub}} n^{\frac{1}{\beta -1}})^{-(\beta -1)} \left( \frac{\beta -2}{\beta -1}\right) ^{\beta -1} (\lambda _{\textrm{hub}}^{-(\beta -1)} - 1) \\&\quad = w^{\beta -1} c_{\textrm{hub}}^{-(\beta -1)}\left( \frac{\beta -2}{\beta -1}\right) ^{\beta -1} (\lambda _{\textrm{hub}}^{-(\beta -1)} - 1) \asymp w^{\beta -1}. \end{aligned}$$

**II.** Then we compute *L* by solving the equation $w_{n-L+1}=c_{\textrm{ldn}}\cdot m^{\lambda _{\textrm{ldn}}}$, which by definition reads

$$\begin{aligned} \frac{\beta -2}{\beta -1} w n ^{\frac{1}{\beta -1}} \cdot (n-L+1)^{-\frac{1}{\beta -1}}&= c_{\textrm{ldn}}\cdot m^{\lambda _{\textrm{ldn}}}. \end{aligned}$$

By multiplying $\left( \frac{\beta -2}{\beta -1} w n ^{\frac{1}{\beta -1}} \right) ^{-1}$ and taking $-(\beta -1)$-power on both sides, we obtain

$$\begin{aligned} n-L+1&= \left( \frac{\frac{\beta -2}{\beta -1} w n ^{\frac{1}{\beta -1}} }{c_{\textrm{ldn}}\cdot m^{\lambda _{\textrm{ldn}}}}\right) ^{\beta -1}\\ L&= n \left[ 1 + n^{-1}- m^{-(\beta -1)\lambda _{\textrm{ldn}}} w^{\beta -1} c_{\textrm{ldn}}^{-(\beta -1)} \left( \frac{\beta -2}{\beta -1}\right) ^{\beta -1}\right] . \end{aligned}$$

By choices of *w* and *m*, we continue

$$\begin{aligned} L&\sim n\left[ 1- n^{-\lambda _{\textrm{ldn}}} c_{\textrm{hub}}^{-(\beta -1)\lambda _{\textrm{ldn}}} w^{\beta -1}c_{\textrm{ldn}}^{-(\beta -1)} \left( \frac{\beta -2}{\beta -1}\right) ^{\beta -1}\right] \\ L&= n\left[ 1- O(n^{-\lambda _{\textrm{ldn}}}w^{\beta -1}) \right] \sim n. \end{aligned}$$

**III.** To count the number of expected low degree nodes among neighbors of an expected hub $i=1,\cdots ,M$, we consider the random variable

$$L_i=\sum _{j=n-L+1}^n X_{ij}$$

which counts the number of neighbors of *i* that are expected low degree nodes. As the edge experiments $X_{ij}$ are independent, Chernoff inequality [CL06, Theorem 2.7] with $\lambda = C\sqrt{\mathbb {E}[L_i]}$ for a free parameter $C>0$ yields

17 $$\begin{aligned} \mathbb {P}(L_i \le \mathbb {E}[L_i] - C\sqrt{\mathbb {E}[L_i]}) \le \exp (-C^2/2). \end{aligned}$$

$$\square $$

Now we prove an auxiliary lemma to estimate the mean expected degree.

#### Lemma 3.9

Under the assumptions of Theorem 3.7, we have

$$\sum _{i=1}^{n}w_i \sim nw.$$

#### Proof of Lemma 3.9

Writing $i_0:=n \left( \frac{w(\beta -2)}{m(\beta -1)}\right) ^{\beta -1}$, we compute

$$\begin{aligned}&\frac{1}{n}\sum _i w_i \sim \frac{1}{n} \int _{i_0}^{i_0+n} c \cdot i^{-\frac{1}{\beta -1}} \textrm{d}i = \frac{1}{n}\frac{\beta -2}{\beta -1} wn^{\frac{1}{\beta -1}} \left[ \frac{i^{1-\frac{1}{\beta -1}}}{1-\frac{1}{\beta -1}}\right] ^{i_0+n}_{i_0}\\&\quad = wn^{\frac{1}{\beta -1}-1} \left[ (i_0+n)^{\frac{\beta -2}{\beta -1}} - i_0^{\frac{\beta -2}{\beta -1}}\right] \\&\quad \sim w. \end{aligned}$$

$$\square $$

Now we compute

$$\begin{aligned} \mathbb {E}[L_i]= \sum _{j=i_0+n-L}^{i_0+n-1} \frac{w_i w_j}{\sum _k w_k}&\sim \frac{w_i}{wn} \int _{i_0+n-L}^{i_0+n} w_j\textrm{d}j= \frac{w_i}{wn}\int _{i_0+n-L}^{i_0+n} \frac{\beta -2}{\beta -1} w n^{\frac{1}{\beta -1}} j^{-\frac{1}{\beta -1}} \textrm{d}j \\&= \frac{w_i}{wn} \frac{\beta -2}{\beta -1} w n^{\frac{1}{\beta -1}} \left[ \frac{j^{1-\frac{1}{\beta -1}}}{1-\frac{1}{\beta -1}}\right] _{i_0+n-L}^{i_0+n}\\&= w_i \left[ (\varepsilon +1)^{\frac{\beta -2}{\beta -1}}- (\varepsilon +1-L/n)^{\frac{\beta -2}{\beta -1}}\right] , \end{aligned}$$

where $\varepsilon := \left( \frac{w(\beta -2)}{m(\beta -1)}\right) ^{\beta -1}=O(w^{\beta -1} n^{-1})$. Using $L\sim n\left[ 1- O(n^{-\lambda _{\textrm{ldn}}}w^{\beta -1}) \right]$ from Step II, we continue

$$\begin{aligned} \mathbb {E}[L_i] \sim w_i \left[ 1+\frac{\beta -2}{\beta -1}\varepsilon +O(\varepsilon ^2)- \left( O(n^{-\lambda _{\textrm{ldn}}}w^{\beta -1}) \right) ^{\frac{\beta -2}{\beta -1}}\right] = w_i \left[ 1+O( n^{-\lambda _{\textrm{ldn}}\frac{\beta -2}{\beta -1}} w^{\beta -2})\right] . \end{aligned}$$

Since $w_i\ge w_{i_0+M-1} = \lambda _{\textrm{hub}} \cdot m \gg \log n$, we put $C = 2\sqrt{\log n}$ in eq. (17) and obtain $\mathbb {P}(L_i \le \mathbb {E}[L_i] - 2\sqrt{\mathbb {E}[L_i]\log n}) \le n^{-2}.$ Now using $\mathbb {E}[ k_i]=w_i=\textrm{Var}[ k_i]$, $M=1$, and $\lambda =2\sqrt{w_i\log n}$, Chernoff upper tail bound [CL06, Theorem 2.6] yields

$$\begin{aligned} \mathbb {P}( k_i\ge w_i + 2\sqrt{w_i\log n})&\le \exp \left( -\frac{4w_i \log n}{2(w_i + 2\sqrt{w_i \log n}/3)}\right) = \exp \left( -\frac{2 \log n}{1 + (2/3)\sqrt{ \log n / w_i})}\right) \\&\le \exp \left( -\frac{2 \log n}{1 + (2/3)}\right) = n^{-6/5}, \end{aligned}$$

where the second inequality follows from our choice that the expected hub degree $w_i\ge \Delta >\log n$.

Hence, with probability $1-M(n^{-2}+n^{-6/5})$, we have simultaneously for each hub $i=i_0,\cdots ,i_0+M-1$ that

$$\begin{aligned} \frac{L_i}{ k_i} > \frac{\mathbb {E}[L_i] - 2\sqrt{\mathbb {E}[L_i]\log n}}{w_i + 2\sqrt{w_i\log n}} =1+O( n^{-\lambda _{\textrm{ldn}}\frac{\beta -2}{\beta -1}} w^{\beta -2}) =: \nu . \end{aligned}$$

With probability $1-O(n^{-1/5})$, our random graph model is $(\Delta ,\delta ,\nu )$-locally star-like. $hfill \square$

#### Shadowing and Typicality on a power-law network

##### Corollary 3.10

Consider *G*-network dynamics (7) satisfying (R1–3), where *G* is a $(\Delta ,\delta ,\nu )$-locally star-like network on $N\gg 1$ nodes with largest degree $\Delta _0$ from the random power-law graph model as in Theorem 3.7, and node dynamics given by iid random iteration of contractions $\varphi _i$ with uniform contraction rate $\lambda \in (0,1)$ and unique stationary measure $m_0$ as in Corollary 3.5. Then, for any error tolerance

$$ \varepsilon \ge \left\{ \alpha \Delta ^{-1/2},4\alpha ^2 \delta \Delta _0^{-1} (1-\lambda )^{-1}\sup _{t\in \mathbb {N}} \Vert h(\theta ^t\omega ,\cdot ,\cdot )\Vert _{C^0} \sup _{t\in \mathbb {N}} |h(\theta ^t\omega ,\cdot ,\cdot )|_{\textrm{Lip}}\right\} , $$

each hub $i\in \mathcal {H}_{\Delta }$ admits $(\varepsilon + \alpha (1-\nu )\sup _{t\in \mathbb {N}} \Vert h(\theta ^t\omega ,\cdot ,\cdot )\Vert _{C^0})$-reduction to $\varphi _{\alpha _i,m_0}$ in Eq. (12) with $\alpha _i=\alpha \frac{\nu _i\kappa _i}{\Delta _0}$, for almost every noise realization $\omega$ and any initial condition $x\in \mathbb {T}^N$, with exceptional asymptotic frequency at most $\rho$ with

$$\rho (\varepsilon ,\omega )= 4 MD(\varepsilon ,\omega )\exp (-\nu \Delta \varepsilon ^2 \alpha ^{-2}c(\omega )),$$

where $D(\varepsilon ,\omega )$ and $c(\omega )$ are the same constants independent of *N* and *x* as in Theorem 2.4.

##### Proof

As in the proof of Corollary 3.5, we take $x_s=x\in \mathbb {T}^N$ and $\omega _s=\omega \in \Omega _*$, verifying the (Typicality) assumption in Theorem 2.18. For the (Shadowing) assumption, we compute

$$\begin{aligned} d_{\mathbb {T}}(x_j^t, \varphi _j(t,\omega ,x_j))&\le d_{\mathbb {T}}(\varphi _j(1,\theta ^{t-1}\omega ,x_j^{t-1}) + \frac{\alpha }{\Delta _0} \sum _{j=1}^N A_{jk} h_{\theta ^{t-1}\omega }(x_j^{t-1},x_k^{t-1}),\\&\quad \varphi _j(1,\theta ^{t-1}\omega )\circ \varphi _j(t-1,\omega ,x_j))\\&\le \frac{\alpha }{\Delta _0} \delta \sup _{t\in \mathbb {N}} \Vert h(\theta ^t\omega ,\cdot ,\cdot )\Vert _{C^0}+ \lambda \cdot d_{\mathbb {T}}(x_j^{t-1}, \varphi _j(t-1,\omega ,x_j))\\&\le \alpha \delta \Delta _0^{-1} \sup _{t\in \mathbb {N}} \Vert h(\theta ^t\omega ,\cdot ,\cdot )\Vert _{C^0}(1+\lambda + \cdot + \lambda ^{t-1}) \\&\le \alpha \delta \Delta _0^{-1} \sup _{t\in \mathbb {N}} \Vert h(\theta ^t\omega ,\cdot ,\cdot )\Vert _{C^0} \frac{1}{1-\lambda },~~~~\forall t\in \mathbb {N}. \end{aligned}$$

We have thus verified almost surely and for each initial condition $x\in \mathbb {T}^N$ the (Shadowing) assumption with shadowing precision $\varepsilon _s = \frac{1}{1-\lambda } \alpha \Delta _0^{-1} \delta \sup _{t\in \mathbb {N}} \Vert h(\theta ^t\omega ,\cdot ,\cdot )\Vert _{C^0}$.

The corollary follows from Theorem 2.18. $\square$

## Data Availability

We do not analyse or generate any datasets, because our work proceeds within a theoretical and mathematical approach.

## Proofs of Theorems A, C and More General Settings

In addition to the hypotheses of Theorems 2.4 and 2.18, if $\mathbb {P}\otimes m^{\otimes N}$-a.e. $(\omega ,x)\in \Omega \times \mathbb {T}^N$ admits shadowing intial data $(\omega _{{s}},x_{{s}})$ satisfying (Shadowing) and (Typicality), then the $(m,\varepsilon )$-reduction holds $\mathbb {P}\otimes m^{\otimes N}$-almost surely. Moreover, if shadowing can be realized in an absolutely continuous way, then one can transfer the measure-theoretic results (ii) Small fluctuation in long time windows and (iii) Gaussian fluctuations from decoupled low degree node dynamics to the coupled network system.

### Definition A.1

*(Absolutely continuous shadowing)* We say that a network system (7) admits *absolutely continous shadowing* if there is a $\mathbb {P}\otimes m^{\otimes N}$-invariant map $\textrm{ACS}:(\omega ,x)\mapsto (\omega _{{s}},x_{{s}})$ for which the network trajectory $x^t$ starting from $\mathbb {P}\otimes m^{\otimes N}$-almost every $(\omega ,x)$ admits shadowing initial data $(\omega _{{s}},x_{{s}})$ satisfying (Shadowing) and (Typicality) as in Theorem 2.18.

We state and prove the result for the star network, assuming $\sup _{\omega \in \Omega } \Vert h(\omega ,\cdot ,\cdot )\Vert _{C^4}\le K$ for some constant $K>0$.

### Theorem A.2

In addition to the hypotheses of Theorem 2.4, assume also that the star network dynamics admits absolutely continuous shadowing map $\textrm{ACS}$ and $\sup _{\omega \in \Omega } \Vert h(\omega ,\cdot ,\cdot )\Vert _{C^4}\le K$ for some constant $K>0$. Then, we have the following.

$\mathrm {(i)}$: **Almost sure reduction in asymptotic time:** for any fixed error tolerance $\varepsilon \ge \max \left\{ \alpha L^{-1/2}, 4\alpha K\varepsilon _s (\alpha L^{-1} K)\right\} ,$ starting from $\mathbb {P}\otimes m^{\otimes N}$-almost every $(\omega ,x)\in \Omega \times \mathbb {T}^N$, the hub behavior (9) admits $\varepsilon$-reduction to $\varphi _{\alpha ,m}$ with exceptional asymptotic frequency at most $\rho$ with $\rho \asymp \varepsilon ^{-1} \exp (-L\varepsilon ^2 \alpha ^{-2}).$
$\mathrm {(ii)}$: **Small fluctuation in long time windows:** there is an exceptional set *E* of initial data in $\Omega \times \mathbb {T}^{N}$ of size $\mathbb {P}\otimes m^{\otimes N}(E) \asymp L^{\kappa } \exp (-L^{1-2\kappa }),~~~~\kappa \in (0,1/2),$ outside of which the fluctuation is well-controlled in time windows $I_{t_0}^T:=\Big \{t_0,\cdots ,t_0+T-1\Big \}$ of exponential length $T\ge \exp ( L^{1-2\kappa })$ starting at any moment $t_0\in \mathbb {N}$ in the sense that $\max _{t\in I_{t_0}^T} |\xi _{m}(\theta ^t\omega , \Phi _{\alpha }(t,\omega ,x))| \le L^{-\kappa } \alpha K^2,~~~~\forall t_0\in \mathbb {N},\forall (\omega ,x)\in \Omega \times \mathbb {T}^N\setminus E;$
$\mathrm {(iii)}$: **Gaussian fluctuations: ** if the coupling map has the form $h(\omega ,x,y)=\phi (x)-\phi (y)+\psi (\omega )$ for some Lipschitz $\phi :\mathbb {T}\rightarrow \mathbb {R}$ and bounded $\psi$, then at any time $t\in \mathbb {N}$, the fluctuation $\xi _{m}(\theta ^t\omega , \Phi _{\alpha }(t,\omega ,x))$ is approximately Gaussian, i.e., $$\begin{aligned}&\forall s\in \mathbb {R}:~~~~ \mathbb {P}\otimes m^{\otimes N}\left\{ (\omega ,x): \xi _{m}(\theta ^t\omega , \Phi _{\alpha }(t,\omega ,x)) \le s\right\} \\&\in \left[ F_L\left( s -c_1\right) -c_2, F_L\left( s+c_1 \right) +c_2\right] ,\end{aligned}$$ where $c_1\asymp \varepsilon _s \left( \alpha L^{-1} \left( 2\Vert \phi \Vert _{C^0}+ \Vert \psi \Vert _{L^{\infty }} \right) \right)$, $c_2\asymp L^{-1/2}$, and $F_L$ denotes the cdf of the normal distribution with zero mean and variance $\alpha ^2 \left[ \int _{\mathbb {T}}\phi ^2\textrm{d}m - \left( \int _{\mathbb {T}}\phi \textrm{d}m\right) ^2\right]$.

Item **(i) Almost sure reduction in asymptotic time** of Theorem A.2 can be proven using Corollaries 3.5 and 3.10. The particular constants are simpler and sharper in the Theorem than in the Corollaries because the coupling function in the introduction example has only one Fourier mode and hence requires no truncation; these detailed calculations are presented in full in [Bia24].

### Proof of Theorem A.2 (ii)

Define

$$\begin{aligned} Q_{{s}} (t,\phi ,\varepsilon _b):= \left\{ (\omega _{{s}}, x_{{s}}): \left| \frac{1}{L}\sum _{j=2}^N \phi \circ \varphi _j(t,\omega _{{s}},x_{{s},j}) - \int _{\mathbb {T}} \phi \textrm{d}m\right| >\varepsilon _b \right\} . \end{aligned}$$

Note $Q_{{s}} (0,\phi ,\varepsilon _b)=\Omega \times B(\varepsilon _b,\phi )$ and hence $m^{\otimes N}(B(\varepsilon _b,\phi ))=\mathbb {P}\otimes m^{\otimes N} (Q_{{s}}(0,\phi ,\varepsilon _b))$. Since *m* is stationary, it follows that $\mathbb {P}\otimes m^{\otimes L}$ is invariant for the *L*-fold skew product

$$(\omega _{{s}},x_{{s},2},\cdots ,x_{{s},N}) \mapsto \left( \theta ^t\omega _{{s}}, \varphi _2(t,\omega _{{s}},x_{{s},2}),\cdots , \varphi _N(t,\omega _{{s}},x_{{s},N})\right) ,$$

and thus $m^{\otimes N}(B(\varepsilon _b,\phi ))=\mathbb {P}\otimes m^{\otimes N} (Q_{{s}}(t,\phi ,\varepsilon _b))$ for all $t\in \mathbb {N}$, where the hub coordinate is free. By Hoeffding, we obtain

$$\mathbb {P}\otimes m^{\otimes N} (Q_{{s}}(t,\phi ,\varepsilon _b)) = m^{\otimes N}(B(\varepsilon _b,\phi ))\le 2\exp \left( -L 2^{-1} \Vert \phi \Vert _{\infty }^{-2} \varepsilon _b^2\right) .$$

For $\phi _n(x):=e^{2\pi i nx}$ we have

$$\mathbb {P}\otimes m^{\otimes N} (Q_{{s}}(t,\phi _n,\varepsilon _b)) \le 2\exp \left( -L 2^{-1} \varepsilon _b^2\right) ,$$

and therefore, in the notation adopted in the proof ot Theorem 2.4, we have

$$\begin{aligned} 4TD\exp (-L2^{-1}\varepsilon _b^2)\ge&\mathbb {P}\otimes m^{\otimes N} \left( \bigcup _{t\in I_{t_0}^T} \bigcup _{1\le |n_2|\le D} Q_{{s}}(t,\phi _{n_2},\varepsilon _b)\right) \\&\quad =\mathbb {P}\otimes m^{\otimes N} \left( \textrm{ACS}^{-1}\bigcup _{t\in I_{t_0}^T} \bigcup _{1\le |n_2|\le D} Q_{{s}}(t,\phi _{n_2},\varepsilon _b)\right) . \end{aligned}$$

For any $(\omega ,x)\notin \textrm{ACS}^{-1}\bigcup _{t\in I_{t_0}^T} \bigcup _{1\le |n_2|\le D} Q_{{s}}(t,\phi _{n_2},\varepsilon _b)$, we have

$$\begin{aligned} \left| \frac{1}{L}\sum _{j=2}^N \phi _{n_2}\circ \varphi _j(t,\omega _{{s}},x_{{s},j}) - \int _{\mathbb {T}} \phi _{n_2}\textrm{d}m\right| \le \varepsilon _b,~~~~\forall t\in I_{t_0}^T,1\le |n_2|\le D, \end{aligned}$$

where $(\omega _{{s}},x_{{s}})=\textrm{ACS}(\omega ,x)$, and hence, by choosing $\varepsilon _b=\frac{30}{\alpha K}\varepsilon$, we obtain

$$|\zeta _{\ell }(t,\omega ,x,\omega _{{s}},x_{{s}})|\le \varepsilon /4,~~~~\forall t\in I_{t_0}^T.$$

In summary, for any fixed

$$\varepsilon \ge 4\alpha K \varepsilon _s (\alpha L^{-1} K),~~~~D(\varepsilon )\asymp \varepsilon ^{-1},~~~~\varepsilon _b=\frac{30}{\alpha K}\varepsilon ,$$

where $D(\varepsilon )=D(\varepsilon ,\omega )$ is now independent of $\omega$ by assumption $\sup _{\omega } \Vert h(\omega ,\cdot ,\cdot )\Vert _{C^4}\le K$, we have

$$|\xi _{m}(\theta ^t\omega ,x^t)| \le \sum _{k=d,h,\ell }|\zeta _k(t,\omega ,x,\omega _{{s}},x_{{s}})| \le \varepsilon /4 + \varepsilon /2 + \varepsilon /4=\varepsilon ,$$

for any $$(\omega ,x)\notin \textrm{ACS}^{-1}\bigcup _{t\in I_{t_0}^T} \bigcup _{1\le |n_2|\le D} Q_{{s}}(t,\phi _{n_2},\varepsilon _b)$$.

Now take $\varepsilon = L^{-\kappa }\alpha K^2$ for some $\kappa \in (0,1/2)$ and $T=\exp \left( L \frac{\varepsilon ^2}{\alpha ^2 K^2}\right) = \exp (L^{1-2\kappa })$, we have

$$ \mathbb {P}\otimes m^{\otimes N} \left( \textrm{ACS}^{-1}\bigcup _{t\in I_{t_0}^T} \bigcup _{1\le |n_2|\le D} Q_{{s}}(t,\phi _{n_2},\varepsilon _b)\right) \le 4TD\exp (-L2^{-1}\varepsilon _b^2)\asymp L^{\kappa } \exp (-L^{1-2\kappa }).$$

This completes the proof of (ii) Small fluctuation in long time windows. $\square$

### Proof of Theorem A.2 (iii)

Fix any time moment $t\in \mathbb {N}$. The fluctuation has the form

$$ \xi ^t = \frac{\alpha }{L} \sum _{j=2}^N \left[ \phi (x_i^t) -\int _{\mathbb {T}}\phi \textrm{d}m \right] . $$

We compare the low degree trajectory $x_i^t$ observed through $\phi$ with the iid random variables

$$\begin{aligned}Y_i^t:\Omega \times \mathbb {T}^N\rightarrow \mathbb {R},~~~~Y_i^t(\omega _s,x_s):= \alpha \left[ \phi \circ \textrm{proj}_i\circ \varphi _i(t,\omega _s,x_s) - \int _{\mathbb {T}}\phi \textrm{d}m\right] ,~~~~i=2,\cdots ,N.\end{aligned}$$

Since $\phi$ is Lipschitz, we obtain that $Y_i^t$ are bounded (from mean value theorem)

$$ |Y_i^t| \le \alpha |\phi |_{\textrm{Lip}}, $$

zero mean

$$\begin{aligned} \mu&:=\mathbb {E}[Y_i^t]=\alpha \left[ \int _{\Omega \times \mathbb {T}^N} \phi \circ \textrm{proj}_i\circ \varphi _i(t,\omega _s,x_s)\textrm{d}\mathbb {P}\otimes m^{\otimes N}(\omega _s,x_s) - \int _{\mathbb {T}}\phi \textrm{d}m\right] \\&=\alpha \left[ \int _{\Omega \times \mathbb {T}^N} \phi \circ \textrm{proj}_i \textrm{d}\mathbb {P}\otimes m^{\otimes N}(\omega _s,x_s) - \int _{\mathbb {T}}\phi \textrm{d}m\right] =0, \end{aligned}$$

finite variance

$$\sigma ^2:=\mathbb {E}[|Y_i^t|^2] = \alpha ^2 \left[ \int _{\mathbb {T}}\phi ^2\textrm{d}m - \left( \int _{\mathbb {T}}\phi \textrm{d}m\right) ^2\right] \le 4|\phi |_{\textrm{Lip}}^2\alpha ^2,$$

and finite third moment

$$\tau :=\mathbb {E}[|Y_i^t|^3] \le 8|\phi |_{\textrm{Lip}}^3\alpha ^3.$$

Now by shadowing estimates and absolutely continuous shadowing map $\textrm{ACS}$, we have

$$\begin{aligned} \mathbb {P}\otimes m^{\otimes N}\left\{ \xi ^t \le s\right\} =&\textrm{ACS}_*\mathbb {P}\otimes m^{\otimes N}\left\{ (\omega ,x): \frac{1}{L} \sum _{j=2}^N \alpha \left[ \phi (x_i^t) -\int _{\mathbb {T}}\phi \textrm{d}m \right] \le s\right\} \\ \le&\mathbb {P}\otimes m^{\otimes N}\left\{ (\omega _s,x_s): \frac{1}{L} \sum _{j=2}^N Y_i^t\le s + |\phi |_{\textrm{Lip}} \alpha \varepsilon _s\right\} \\ =&\mathbb {P}\otimes m^{\otimes N}\left\{ (\omega _s,x_s): \frac{1}{\sigma \sqrt{L}} \sum _{j=2}^N Y_i^t\le (s + |\phi |_{\textrm{Lip}} \alpha \varepsilon _s) \frac{\sqrt{L}}{\sigma }\right\} \\ \le&\Phi _{\mathcal {N}(0,1)}\left( (s+ |\phi |_{\textrm{Lip}}\alpha \varepsilon _s )\frac{\sqrt{L}}{\sigma }\right) + \frac{3\tau }{\sigma ^3}\sqrt{L} \\ =&\Phi _{\mathcal {N}(0,\sigma ^2/L)}\left( s+ |\phi |_{\textrm{Lip}}\alpha \varepsilon _s \right) + \frac{3\tau }{\sigma ^3\sqrt{L} } \end{aligned}$$

where the second approximation follows by Berry-Esseen [Dur19, Theorem 3.4.9], the last equality follows from the Gaussian cdf relation $\Phi _{\mathcal {N}(\mu ,\sigma ^2)}(s) = \Phi _{\mathcal {N}(0,1)}\left( \frac{x-\mu }{\sigma }\right)$, and $\sigma ^2 =\alpha ^2 \left[ \int _{\mathbb {T}}\phi ^2\textrm{d}m - \left( \int _{\mathbb {T}}\phi \textrm{d}m\right) ^2\right]$, $\varepsilon _s = \varepsilon _s \left( \alpha L^{-1} \left( 2\Vert \phi \Vert _{C^0}+ \Vert \psi \Vert _{L^{\infty }} \right) \right)$. The lower bound can be obtained similarly. $\square$

### Proof of Theorem A

Since the coupling function (3) has only one Fourier mode, the Fourier truncation in Lemma 2.13 is unnecesary and the bad set estimates in Lemma 2.14 reduce to one bad set only.

Item (i) follows by a simplified version of Theorem 2.4 together with the uniform typicality Theorem 3.3. The shadowing precision $\varepsilon _s=\frac{17\alpha }{3L}$ can be calculated as in Corollary 3.5. We choose $\varepsilon _b=\frac{\varepsilon }{2\alpha }$ so that the shadowing and bad set arguments each contribute $\varepsilon /2$ to the reduction estimate.

Item (ii) follows from a similar argument to Theorem A.2 Item (ii) with $\varepsilon _b=L^{-\kappa }\sqrt{6}$ and $\varepsilon _s=\frac{17\alpha }{3L}$.

Item (iii) follows from Theorem A.2 Item (iii) with $\phi (x)=\sin 2\pi x$, $\psi (\omega )=\omega /3.6$.

### Proof of Theorem C

Item (i) follows by a simplified version of Theorem 2.18 together with the uniform typicality Theorem 3.3. The non ldn contribution from Lemma 2.20 is $|\zeta _{i,c}|\le \frac{17}{6}\alpha (1-\nu )\le \varepsilon /3$, by our choice of $\varepsilon$. The shadowing precision $\varepsilon _s=\frac{17\alpha \delta }{3\Delta }$ can be calculated as in Corollary 3.10. We choose $\varepsilon _b=\frac{\varepsilon }{3\alpha }$ so that the shadowing and bad set arguments each contribute $\varepsilon /3$ to the reduction estimate.

Item (ii) follows from a similar argument to Theorem A.2 Item (ii) with $\varepsilon _b=\Delta ^{-\kappa }\sqrt{6}$ and $\varepsilon _s=\frac{17\alpha \delta }{3\Delta }$.

Item (iii) follows from a similar argument to Theorem A.2 Item (iii) with $\phi (x)=\sin 2\pi x$, $\psi (\omega )=\omega /3.6$. The correction constants $s_1,s_2$ need to account for non ldn contribution.

$$s_1 : = \alpha _i \frac{1}{\nu _i k_i}\sum _{j\in \mathcal {N}_i\cap \mathcal {L}_{\delta }} (\sin 2\pi x_i^t-\sin 2\pi y_j^t ) + \alpha \frac{1}{\Delta _0} \sum _{j\in \mathcal {N}\setminus \mathcal {L}_{\delta }} h_{\omega _i^t}(z_i^t,x_j^t).$$

Using $|h|\le 2+ \frac{3}{3.6} = \frac{17}{6}$ and $\varepsilon _s = \frac{17}{3}\alpha \frac{\delta }{\Delta }$, we have

$$|s_1| \le \alpha _i 2\pi \varepsilon _s + \alpha \Delta _0^{-1} (1-\nu _i) k_i \frac{17}{6} = \alpha _i 2\pi \frac{17}{3}\alpha \frac{\delta }{\Delta } + \alpha \Delta _0^{-1} (1-\nu _i) k_i \frac{17}{6},$$

which simplifies to

$$|s_1| = O(N^{-\frac{1-\lambda _{\textrm{ldn}}}{\beta -1}} + N^{-\lambda _{\textrm{ldn}} \frac{\beta -1}{\beta -2}} w^{\beta -2}).$$

Lastly, the Berry-Esseen correction, similar to that in Theorem A.2 Item (iii), reads

$$s_2 := \frac{C \int _0^1|\sin 2\pi x|^3\textrm{d}x}{2^{-3/2} \sqrt{\nu _i k_i}} = O(N^{-\frac{1}{2\beta -2}}),~~~~\forall i\in \mathcal {H}_{\Delta }.$$
